# The Role of *piRNA*-Mediated Epigenetic Silencing in the Population Dynamics of Transposable Elements in *Drosophila melanogaster*

**DOI:** 10.1371/journal.pgen.1005269

**Published:** 2015-06-04

**Authors:** Yuh Chwen G. Lee

**Affiliations:** Department of Ecology and Evolution, University of Chicago. Chicago, Illinois, United States of America; Stanford University, UNITED STATES

## Abstract

The *piwi*-interacting RNAs (*piRNA*) are small RNAs that target selfish transposable elements (TEs) in many animal genomes. Until now, *piRNAs’* role in TE population dynamics has only been discussed in the context of their suppression of TE transposition, which alone is not sufficient to account for the skewed frequency spectrum and stable containment of TEs. On the other hand, euchromatic TEs can be epigenetically silenced via *piRNA*-dependent heterochromatin formation and, similar to the widely known “Position-effect variegation”, heterochromatin induced by TEs can “spread” into nearby genes. We hypothesized that the *piRNA*-mediated spread of heterochromatin from TEs into adjacent genes has deleterious functional effects and leads to selection against individual TEs. Unlike previously identified deleterious effects of TEs due to the physical disruption of DNA, the functional effect we investigated here is mediated through the epigenetic influences of TEs. We found that the repressive chromatin mark, H3K9me, is elevated in sequences adjacent to euchromatic TEs at multiple developmental stages in *Drosophila melanogaster*. Furthermore, the heterochromatic states of genes depend not only on the number of and distance from adjacent TEs, but also on the likelihood that their nearest TEs are targeted by piRNAs. These variations in chromatin status probably have functional consequences, causing genes near TEs to have lower expression. Importantly, we found stronger selection against TEs that lead to higher H3K9me enrichment of adjacent genes, demonstrating the pervasive evolutionary consequences of TE-induced epigenetic silencing. Because of the intrinsic biological mechanism of *piRNA* amplification, spread of TE heterochromatin could result in the theoretically required synergistic deleterious effects of TE insertions for stable containment of TE copy number. The indirect deleterious impact of *piRNA*-mediated epigenetic silencing of TEs is a previously unexplored, yet important, element for the evolutionary dynamics of TEs.

## Author Summary

The *piwi-*interacting RNAs (*piRNAs*) are small RNAs that can suppress the expression of selfish transposable elements (TEs) in many animal genomes. One mechanism by which *piRNAs* silence TEs is through the formation of heterochromatin, which is condensed chromatin and generally associated with repressed gene expression. Several functional studies have demonstrated that *piRNA-*mediated heterochromatin of TEs can spread to adjacent genes. We hypothesized that this spread of TE-induced heterochromatin influences the function of adjacent genes, ultimately resulting in selection against individual TEs. Consistent with our hypothesis, we found that sequences and genes adjacent to TEs are enriched in heterochromatic marks. We determine that this TE-induced variation in epigenetic status is probably *piRNA-*dependent and that this change in chromatin state influences the expression levels of adjacent genes. Importantly, TEs that lead to higher heterochromatin enrichment of adjacent genes are more strongly selected against, demonstrating the evolutionary consequences of TE-induced epigenetic silencing. In contrast to previously studied deleterious impacts of TEs, which depend on TEs’ physical disruptions of DNAs, our proposed functional effect of TEs is mediated through their epigenetic influence. Our study suggests that the *piRNA*-dependent epigenetic impact of TEs may play an important role in the evolutionary dynamics of TEs.

## Introduction

Transposable elements (TEs) are genetic elements that increase their copy number in the host genome by copying themselves to new genomic locations. Despite reported incidences of potentially adaptive TEs [1–6], the majority of TEs are considered deleterious to their host and widely viewed as “genomic parasites”. TE insertions can disrupt sequence and function of host genetic elements [7]. Additionally, ectopic recombination between nonhomologous TE copies leads to potentially deleterious chromosomal rearrangements [8–12]. Although deleterious impacts of TE insertions are broadly appreciated, our picture for the functional and evolutionary mechanisms of TEs containment in natural populations is still incomplete.

Because of the replicative nature of most transposition mechanisms of TEs, it is critical to understand the evolutionary forces counterbalancing the constant increase of TEs. Theoretical models demonstrate that the TE copy number in an outbreeding population reaches an equilibrium when the increase in TE copy number via transposition is counterbalanced by the removal of TEs [13,14]. Regulating the transposition rate such that it equals the excision rate of TEs is one possible mechanism of achieving equilibrium. This, however, is not supported by empirical observations (reviewed in [15]). In addition, theoretical studies have shown that transposition rate has only a minimal impact on the predicted frequency spectrum of TEs [13,14]. Regulation of transposition rate is unlikely to account for the observed low population frequency of most TEs in outbreeding populations such as *Drosophila* ([4,16–19], reviewed in [15]). Instead, both theoretical analyses and empirical results support that selection against deleterious impacts of TEs plays a primary role in the evolutionary dynamics of TEs [15,20,21]. Without regulated TE transposition, selection alone can result in an equilibrium of TE copy number and lead to the skewed frequency spectrum of TEs in natural populations.

Most previous work on the deleterious effects of TEs has centered around consequences of TE-mediated physical disruption of genomic DNA, such as TEs’ insertion into functional elements and ectopic recombination between nonhomologous TEs. The exploration of the potential functional and evolutionary consequences of TEs’ epigenetic impacts have been limited [22]. In *Drosophila*, a class of small interfering RNA, *piwi-*interacting RNAs (*piRNAs*), are enriched for TE sequences and post-transcriptionally regulate TE transposition in the germline [23–26]. In addition, *piRNAs* can suppress TE transposition by inducing heterochromatic formation of euchromatic TEs in the germline [27–31] as well as in larval and adult somatic tissues [32,33]. Constitutive pericentric and telomeric heterochromatin “spreads” and usually results in stochastic silencing of the nearby genes, a phenomenon known as “Position-effect variegation” (PEV [34], reviewed in [35–37]). Similarly, *piRNA-*mediated heterochromatin of euchromatic TEs was shown to spread into adjacent host genes by using reporter constructs [32]. The resulting perturbation of host gene expression due to the spread of heterochromatin from adjacent TEs probably has deleterious consequences. These TEs are expected to be removed by selection even if they do not generate physical disruptions of the DNA, leading to a skewed frequency spectrum of TEs [20].

**Fig 1:**
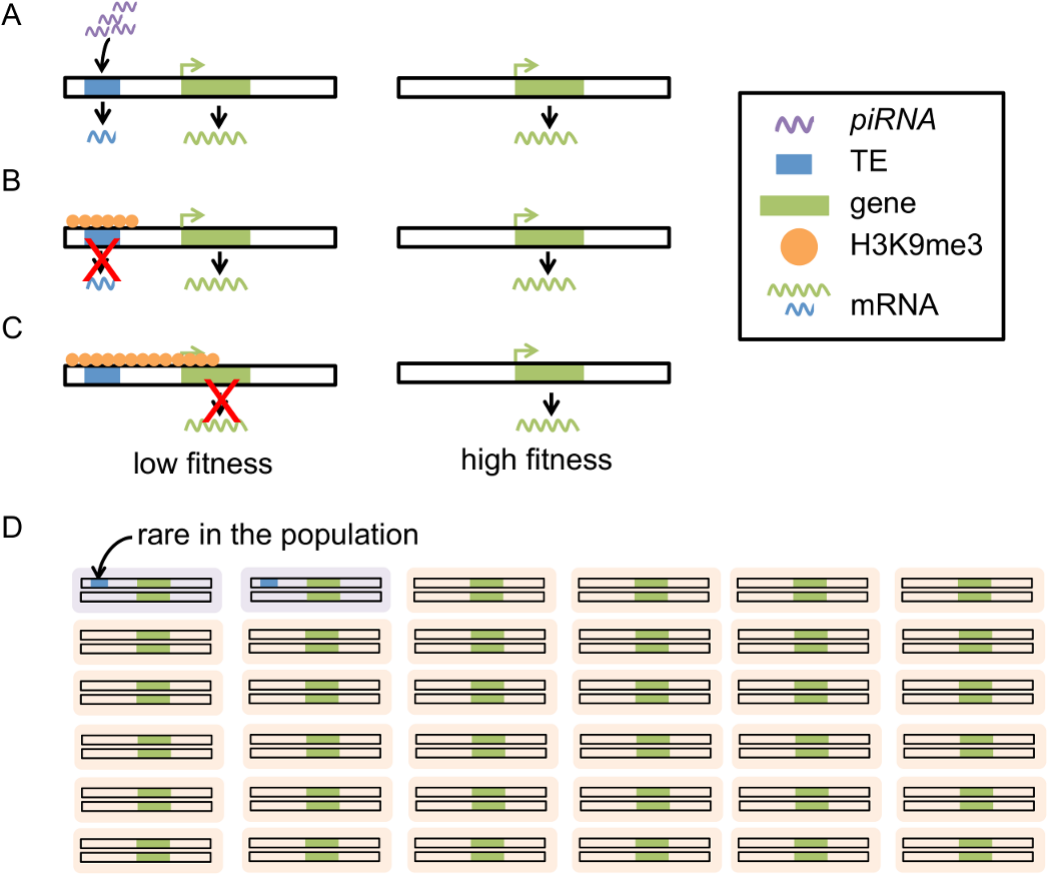
Proposed model for the evolutionary impacts of *piRNA-*mediated epigenetic silencing of TEs. *piRNAs* target TEs that have complementary sequences (A), leading to installation of heterochromatic marks (*i*.*e*. H3K9me) on TE sequences (B). The boundaries of heterochromatin are generally dynamic and heterochromatin induced by TEs can “spread” into adjacent sequence. The spread of heterochromatin from TEs into genes could lead to lowered gene expression, impairing the function of genes (C). Individuals with these TE insertions will have lower fitness (purple in D) than individuals without (orange in D). TEs with this mutational impact are expected to be selected against, remaining rare in the population (D).

Despite detailed functional studies investigating the epigenetic influence of TEs on surrounding sequences, the influence of naturally occurring TEs on the chromatin states and functions of nearby genes has not been explored on a local or genomic scale in *Drosophila*. The connection between TE-induced epigenetic changes and the evolutionary genomic consequences of TE insertions is also lacking. In this study, we tested our hypothesis that the spread of *piRNA-*mediated heterochromatin of TEs to adjacent genes is deleterious and represents an important force shaping the population dynamics of TEs (Fig 1).

Throughout our study, we used the distribution of histone modifications as an index for the chromatin state of a region. Within the nucleus, DNA is wrapped around core histones (H2A, H2B, H3, and H4) to form nucleosomes, the fundamental unit of chromatin. Modifications of histones, such as methylation and acetylation, at different positions of the core histones have been associated with biological consequences, particularly gene expression states (reviewed in [38]). For instance, tri-methylation of histone H3 lysine 4 (H3-K4me3) is enriched at transcription start sites and found to correlate positively with gene expression levels [39,40]. The methylation of histone H3 lysine 9 (H3K9me), particularly di- and tri- methylations, is generally regarded as “repressive” mark of the chromatin and is found enriched in the heterochromatic regions of the *Drosophila* genome [38–41]. We focused on the genomic variation in the level of H3K9me, which allows us to relate TE-composition to the heterochromatic states of sequences.

## Results

### Distance from intergenic TE insertion correlates negatively with H3K9me3 enrichment

Previous local studies indicated that the density of heterochromatic marks is elevated in sequences adjacent to TEs [32], which our hypothesis relies on (Fig 1B and 1C). To confirm this finding with naturally occurring TEs on a genomic scale, we used the genome annotation of the *D*. *melanogaster* reference genome (the *Drosophila* genome with the best annotated TEs), and the modEncode H3K9 tri-methylation (H3K9me3) data of the same strain at nine developmental time stages (six embryonic stages, two larval stages, and one pupal stage, [40]). These experiments were performed using whole animals, which consist of heterogeneous tissues and cell types. Histone modifications could significantly vary across cell types [42] and it is difficult to interpret the modifications of samples consisting of heterogeneous tissues and/or cell types as binary states [43,44]. Accordingly, we analyzed H3K9me3 data quantitatively, using the average read density of H3K9me3 for our following analyses (see Materials and Methods).

**Fig 2:**
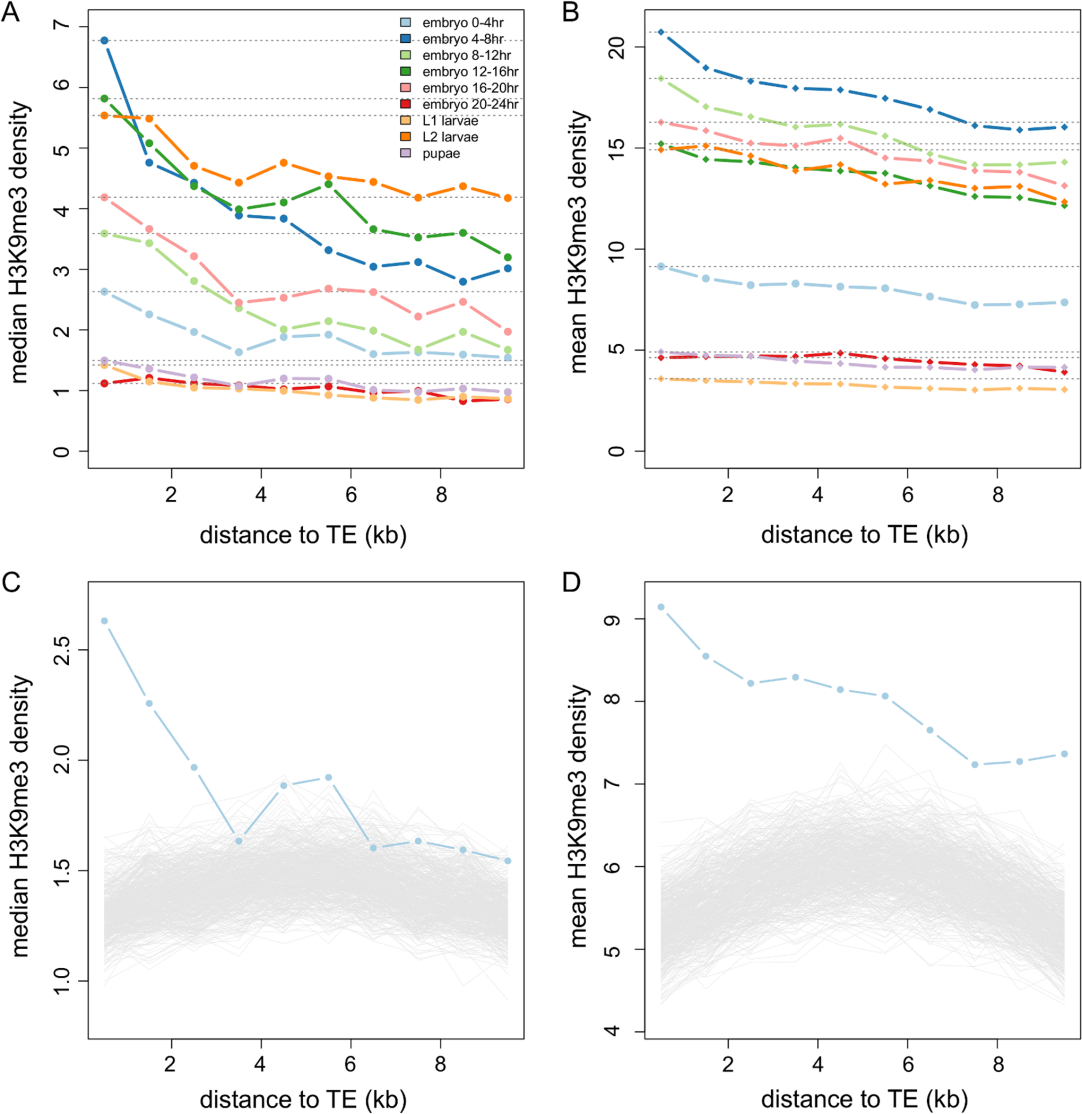
The decay of H3K9me3 density of intergenic sequences adjacent to TEs. The median (A) and mean (B) of H3K9me3 density in nonoverlapping 1kb windows of intergenic sequences decreases as the windows are farther from TEs. Different colors are for different developmental stages: embryo 0–4 hr (light blue), embryo 4–8 hr (blue), embryo 8–12 hr (light green), embryo 12–16 hr (green), embryo 16–20 hr (pink), embryo 20–24 hr (red), L1 larvae (yellow), L2 larvae (orange), and pupae (purple). Dashed lines are the median/mean of the H3K9me3 density in the window closest to TEs. The observed median (C) and mean (D) H3K9me3 densities of windows adjacent to TEs are higher than those adjacent to 1,000 sets of randomly chosen TE-size sequences (gray lines), particularly for windows that are closer to TEs. Results of 0–4 hr embryos are shown here. Note that the scales of y-axis are different between (A) and (B) and between (C) and (D).

To investigate whether the TE-induced heterochromatin spreads beyond TEs, we first examined the enrichment of heterochromatic marks adjacent to TEs. We did this by estimating the H3K9me3 density at sequences that are 10kb upstream and downstream of euchromatic TEs. We only considered 10kb sequences that are entirely within intergenic regions to avoid the potential influences from functional elements (genes and regulatory sequences), which are generally depleted of “silent” marks (such as H3K9me) and enriched with other “active” histone modifications ([39,40], reviewed in [38]). We observed that the H3K9me3 density of 1kb nonoverlapping windows decreases with increasing distance from TEs (Fig 2A and 2B). The windows closest to a TE (0-1kb and 1-2kb) have significantly higher H3K9me3 density than the most distant window analyzed (9-10kb, *Mann-Whitney U test*, *p <* 0.011; S1 Table) for all developmental stages. For some embryonic stages, the significant difference in H3K9me3 enrichment is still observable for windows that are even farther from TEs (S1 Table).

We compared our observations to null genomic expectations by randomly choosing genomic segments that are of the same sizes and the same large-scale chromosome locations as those of TEs and investigating the decay in H3K9me3 enrichment near these “TE-size” sequences (see Materials and Methods). The median (Fig 2C) and mean (Fig 2D) H3K9me3 of windows adjacent to TEs are indeed higher than those of random sequences for most developmental stages (see S1 and S2 Figs for all developmental stages). Our genomic observations support predictions from functional data [32] that the presence of TEs influences the chromatin status of adjacent sequences.

### Genes adjacent to TEs have higher H3K9me3 than other genes

**Fig 3:**
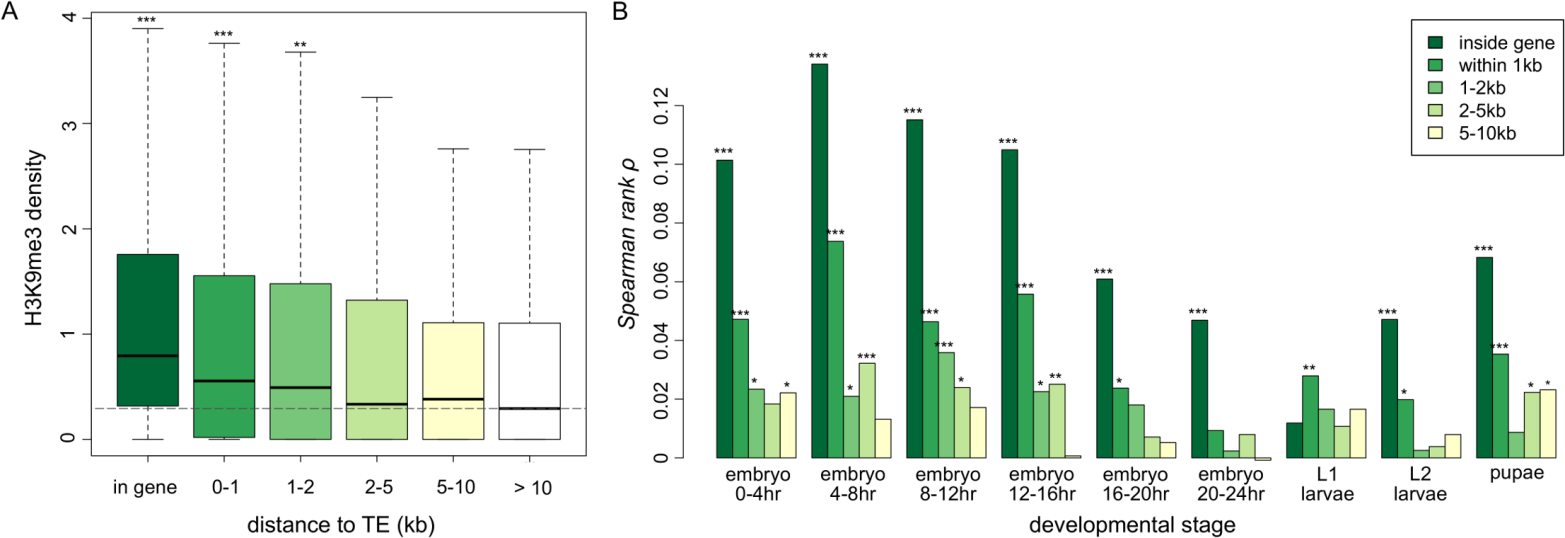
The associations between H3K9me3 density of genes and their neighborhood TE content. (A) Boxplots for the H3K9me3 density of genes that are of different distance from TEs. The H3K9me3 density of genes that have at least one TE in the introns of the gene (in gene), within 1kb, 1-2kb, 2-5kb, and 5-10kb upstream/downstream from the gene are significantly lower than those of genes that have no TE 10kb upstream/downstream (whose median of H3K9me3 density is shown as a dashed line). Numbers of genes in each category are 588 (in gene), 579 (within 1kb), 323 (1-2kb), 859 (2-5kb), 1162 (5-10kb), and 8072 (no TE in 10kb). All comparisons are against genes without TEs in 10kb upsteam/downstream. Result of 0–4 hr embryo is shown. (B) The *spearman rank* correlation coefficients between H3K9me3 density of a gene and the number of adjacent TEs within a specific window decrease as the distance between genes and TEs increases. The median, mean, and maximum number of TEs in specific windows are (1) in gene: 0 (median), 0.0523 (mean) and 8 (maximum), (2) 0-1kb: 0 (median), 0.0555 (mean), and 4 (maximum), (3) 1-2kb: 0 (median), 0.0341 (mean), 2 (maximum), (4) 2-5kb: 0 (median), 0.101 (mean), and 5 (maximum), and (5) 5-10kb: 0 (median), 0.158 (mean), and 7 (maximum). Notations for *p-values* are * (*p* < 0.05), ** (*p* < 0.01), and *** (*p* < 0.001).

In order to support our hypothesis, it is critical to demonstrate that the spreading of TE-induced heterochromatin also influences the chromatin states of neighboring genes in addition to intergenic sequences (Fig 1C). Using the same H3K9me3 data of the reference strain at nine developmental stages, we contrasted the enrichment of H3K9me3 marks in euchromatic genes that have TEs inside their introns (in gene), in 1kb, 1-2kb, 2-5kb, 5-10kb upstream/downstream of the gene to those of genes that have no TEs within 10kb upstream/downstream. Similar to our observation for intergenic sequences, we found genes with TEs nearby have significantly higher H3K9me3 density than genes without TEs nearby. H3K9me3 enrichment decreases as the windows move further away from TEs (Fig 3A for 0–4 embryo, see S3 Fig for all developmental stages). Consistently, we found significant negative correlations between a gene’s H3K9me3 density and its distance from the nearest TE (*Spearman rank ρ* = -0.189 ~ -0.040, *p <* 0.05 for L1 and L2 larvae and *p <* 10^–3^ for all other developmental stages, S2 Table). In addition, the number of TEs in bins further away from genes is less correlated with a gene’s H3K9me3 density (Fig 3B). These observations suggest that genic H3K9me3 enrichment correlates with a gene’s surrounding TE composition, both in terms of TE number and distance between genes and TEs.

Our observations are not merely driven by genes that are in highly heterochromatinized genomic regions. Excluding genes in H3K9me3 enriched regions in another strain of the modEncode project (at embryonic or larval stage of Oregon-R strain) or in genomic regions that are classified as state 7 (highly enriched with H3K9me2/3) or state 8 (moderately enriched with H3K9me2/3) in either S2 or BG3 cell lines [39] gave similar results (S4–S6 Figs for comparisons of H3K9me3 density of genes that are of different distance from TEs; S7–S9 Figs for correlations between H3K9me3 density of genes and the nearby number of TEs).

It is important to examine whether the observed associations between the H3K9me3 enrichment of a gene and its neighborhood TE content can be attributed to other confounding factors. In *D*. *melanogaster*, TEs are known to accumulate in genomic regions with low recombination rate [16,18,45–48] and potentially regions with low gene density (although see [47]). We found that genic H3K9me3 density is negatively correlated with local gene density (*Spearman rank ρ* = -0.217 ~ -0.097, *p <* 10^–16^ for all developmental stages) and weakly correlated with its local recombination rate (*Spearman rank ρ* = -0.040 ~ -0.021, *p <* 0.05 for all but one developmental stage). However, we still observed significant positive correlations between a gene’s H3K9me3 density and its adjacent TE numbers using partial correlation analyses (S10 Fig for controlling for gene density and S11 Fig for controlling for local recombination rate), suggesting that neither the effects of recombination rate nor gene density can solely account for our observations.

### Genic H3K9me3 enrichment is positively correlated with *piRNA* targeting of adjacent TEs

Heterochromatin formation of TEs in somatic tissues is observed to be *piRNA-*dependent [32]. We hypothesized that TEs targeted by large number of *piRNAs* are likely to be transcriptionally silenced, and, consequently, more likely to influence the chromatin states of nearby genes. We estimated the *piRNA* density of TEs [the average (per bp) number of *piRNAs* mapped to TEs; see materials and methods] in the reference genome using *ovarian piRNA* sequence of the reference strain (generated by Shpiz *et al*. [49]). It is worth noting that the H3K9me3 and *piRNA* data were collected using different types of tissues [embryos, larvae, and pupae (H3K9me3) vs ovary (*piRNA*)]. Nevertheless, embryonic *piRNAs* are known to be maternally deposited [50,51].

Consistent with our hypothesis that TEs targeted by *piRNAs* are more likely to influence the chromatin states of adjacent genes, we observed significant positive correlations between the H3K9me3 density of a gene at an early embryonic stage (0–4 hour embryo) and the ovarian *piRNA* density of its nearest TE [*Spearman rank ρ* = 0.043, *p* = 0.016 (sense *piRNA* vs H3K9me3); *Spearman rank ρ* = 0.084, *p <* 10^–5^ (antisense *piRNA* vs H3K9me3)]. As expected, these correlations are stronger for gene-TE pairs that are closer to each other than those that are farther apart [sense TE *piRNA vs*. genic H3K9me3: *Spearman rank ρ* = 0.081 (*p =* 0.008) for short distances between a gene and its nearest TE; antisense TE *piRNA vs*. genic H3K9me3: *Spearman rank ρ* = 0.131 (*p* < 10–4) for short, 0.063 (*p =* 0.038) for intermediate, and 0.057 (*p =* 0.061) for long distances between a gene and its nearest TE; see S12 Fig]. It is worth noting that, although significant, the observed correlations between embryonic H3K9me3 density and ovarian *piRNA* density of nearest TEs are rather weak. This might be due to the differences between ovarian and embryonic *piRNAs*. Future studies using embryonic *piRNAs* could further address this issue. Similar to previous studies [49,52,53], we identified a small fraction of *piRNAs* that target host genes (S3 Table). However, we did not observe similar *positive* correlations between H3K9me3 density and ovarian *piRNA* density of *a gene* (S3 Table), suggesting that our observed higher H3K9me3 of genes with TE nearby is more attributable to the spreading of H3K9me3 from transcriptionally silenced TE instead of by *piRNAs’* transcriptional silencing directly at the host genes.

### TE insertions lead to reduced expression of adjacent genes

H3K9me has been widely associated with the silencing of gene expression (reviewed in [38]). Indeed, we observed significant negative correlations between the H3K9me3 density of a gene and its expression level at the corresponding developmental stage (*Spearman rank ρ* = -0.248 ~ -0.029, *p <* 0.05 for all genes or only genes having TE within 10kb, S4 Table). Given our observation that genes with adjacent TEs have higher H3K9me3 density than genes without, transcriptional output from the former genes should be relatively lower. Indeed, in the majority of developmental stages, genes with TEs nearby have significantly lower expression levels than genes without TEs (S5 Table).

The analysis reported above cannot distinguish the causal relationship between a gene’s expression level and its nearby TE content. Our observations are consistent with either TE insertions leading to reduced expression of adjacent genes or TEs preferentially inserting near genes with low expression. To genomically assess these alternative scenarios, we compared the expression of alternative “alleles” (with or without TEs) of a gene, using TE polymorphism [19] and adult expression data [54] from a North American population. We compared the average expression rank of alleles with at least one TE within a window (“with TE”) to those of “without TE” alleles and performed permutation to assess significance. There is an excess of genes that have significantly (permutation *p-value <* 0.05) *lower* expression of “with TE” alleles than those of “without TE” alleles for all window sizes chosen and for both adult female and adult male (*Fisher’s Exact*, *p* < 0.05, *odds ratio* = 1.46~2.60, Table 1; see S6 and S7 Tables for results of all genes). Because female and male individuals from the same strain have the same type of allele, it is expected that genes having differential expression between “with TE” and “without TE” alleles in one sex should also show the same pattern in another sex. Indeed, we found an excess of genes that are differentially expressed between “with TE” and “without TE” alleles in both female and male (S8 Table). In short, alleles with nearby TEs are more likely to have lower expressions than their homologs without TEs. It is worth noting that the disruption of regulatory sequences by TE insertion could also contribute to this observation [55].

**Table 1:** Differences in expression levels between alleles with and without TEs.

	**female**							
	**median of rank diff.** ^1^		**number of genes**					
**window size with TE**	**significant**	**in- significant**	**significant** ^2^	**in- significant**	**expected significant** ^3^	**prop. of significant**	***FET p-value***	**odds ratio**
**in gene**	732.3	4.5	41	307	17	0.1178	1.42E-03	2.5969
**1kb**	798.0	14.0	48	421	23	0.1023	2.82E-03	2.2091
**2kb**	771.5	13.7	50	528	29	0.0865	1.92E-02	1.7918
**5kb**	712.6	1.1	78	887	48	0.0808	7.32E-03	1.6795
**10kb**	593.2	-6.2	136	1714	93	0.0735	3.21E-03	1.5160
	**male**							
	**median of rank diff.**		**number of genes**					
**window size with TE**	**significant**	**in- significant**	**significant**	**in- significant**	**expected significant**	**prop. of significant**	***FET p-value***	**odds ratio**
**in gene**	725.9	1.8	40	308	17	0.1149	2.11E-03	2.5254
**1kb**	668.3	26.6	51	418	23	0.1087	9.59E-04	2.3638
**2kb**	790.6	14.0	53	525	29	0.0917	8.07E-03	1.9101
**5kb**	613.4	1.9	79	886	48	0.0819	5.70E-03	1.7029
**10kb**	657.8	-2.2	131	1719	93	0.0708	8.53E-03	1.4561

1. Median for the differences in expression rank between alleles with and without TEs. Rank differences were calculated as the mean expression rank of “with TE” alleles minus that of “without TE” alleles. A positive rank difference means “with TE” alleles have larger expression rank (and thus lower expression) than “without TE” alleles.

2. The number of genes whose “with TE” alleles have significantly (permutation *p-value* < 0.05) larger expression rank (i.e. lower expression) than “without TE” alleles.

3. The expected number of genes whose “with TE” alleles have significantly larger expression rank (i.e. lower expression) than “without TE” alleles under the null hypothesis with 5% false positive rate.

### TEs adjacent to genes with high H3K9me3 have lower population frequencies

**Fig 4:**
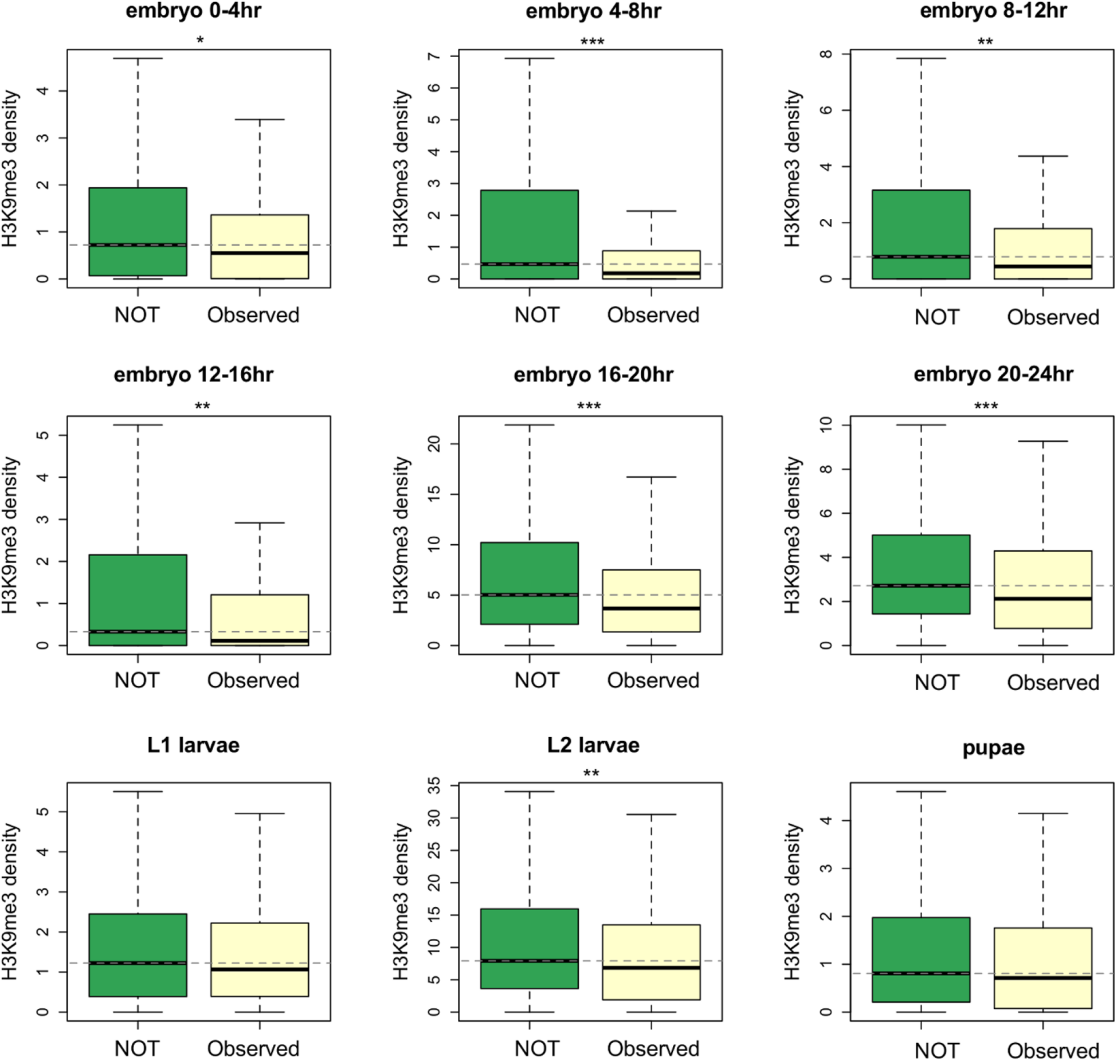
Comparisons of the H3K9me3 density for genes whose nearest TEs have different population frequencies. TEs that are observed in the North American *D*. *melanogaster* population (Observed) have higher population frequencies than those that are not observed in the population (NOT). Genes whose nearest TEs are observed in the population have significantly lower H3K9me3 density. Dashed lines show the median of H3K9me3 density for genes whose nearest TE are not observed in the population. Numbers of genes included in the analysis are 297 (for Observed) and 491 (for NOT). Notations for *p-values* are * (*p* < 0.05), ** (*p* < 0.01), and *** (*p* < 0.001).

Most TE insertions in the Drosophila population are polymorphic [15,18,19,48] and individual TE insertions present at lower population frequencies are expected to have experienced stronger selection removing them. Our hypothesis predicts that TE insertions inducing higher heterochromatic mark enrichment of neighboring genes should have larger deleterious impacts, are more likely removed from the population by selection, and as a result should segregate at lower population frequencies than other TEs. We classified *reference TEs* into those that are observed (high-frequency TEs) and not observed (low-frequency TEs) in a North American population [19]. In the reference genome, genes whose nearest TE occurs at low frequency (not observed in the North American population) have higher median H3K9me3 density than those near high-frequency TEs (*Mann-Whitney U test*, *p* < 0.01 for all developmental stages except for L1 larvae and pupae, Fig 4). Furthermore, we found significant negative correlations between a gene’s H3K9me3 density and the population frequency of its nearest TE in the North American population (*Spearman rank ρ =* -0.152 ~ -0.083, *p <* 0.01 for all developmental stages except for L1 larvae and pupae, S9 Table). Unless multiple independent insertions at the same site happen frequently, the alternative hypothesis that H3K9me3 enrichment inhibits insertions cannot explain our observed negative associations between genic H3K9me3 enrichment and population frequencies of their nearest TEs. Indeed, even though insertion site preference has been reported for several TE families [56–58], multiple independent insertions from the same TE family at the same genomic location have not been documented in *Drosophila*.

Another prediction of our model is that TEs inducing heterochromatin spreading at more developmental stages are expected to have larger cumulative deleterious impacts. Indeed, we found a significant negative correlation between the numbers of developmental stages during which a gene is enriched for H3K9me3 (top 10% genome-wide) and the population frequency of its nearest TE (*Spearman rank ρ =* -0.159, *p <* 10^–5^). This association is not merely driven by genes that have low (below first quantile) H3K9me3 density in all developmental stages, because a significant negative correlation was still observed after removing these genes (*Spearman rank ρ =* -0.160, *p <* 10^–5^). Removing genes with high H3K9me3 enrichment in another strain or cell lines (see Materials and Methods) and using a different threshold to categorize “high H3K9me3 genes” both gave consistent results [*Spearman rank ρ =* -0.148, *p<* 10^–3^ (exclude genes with high H3K9me3 in Oregon-R), *ρ* = -0.166, *p<* 10^–4^ (exclude genes in 7 or 8 chromatin state in cell lines), and -0.112, *p* = 0.002 (use top 25% genome-wide threshold)].

Compared to young and actively transposing TE families, old TE families were found to have lower *piRNA* density [59]. According to our model, we expect old TE families to be less likely be epigenetically silenced and influence the chromatin states of their adjacent genes. Members of old TE families are usually observed at higher population frequencies due to the age of their insertion events. This has the potential to confound our observations. We performed ANOVA that jointly considers the effect of the population frequency and family identity of nearest TEs on genic H3K9me3 enrichment. Our analysis still found that whether a gene’s nearest TE is observed in the North American population or not significantly contributes to the variation of genic H3K9me3 density in the majorities of developmental stages (S10 Table), suggesting that variation in TE age across families is unlikely the sole factor driving our observation.

Our observed negative associations between H3K9me3 of a gene and the frequency of the nearest TE could be confounded by the longer length of TEs adjacent to genes enriched with H3K9me3 (*Spearman rank ρ* between a gene’s H3K9me3 and the length of nearest TE, 0.036 ~ 0.118, *p <* 0.05 for all developmental stages). Longer TEs are expected to undergo ectopic recombination more frequently, more likely to be selected against, and thus be at lower population frequencies, a theoretical result which has been supported by empirical data [9,10,48,60]. However, given equal *piRNA* density, longer TEs represent larger *piRNA* targets and are more likely to be silenced as well as to have deleterious epigenetic impacts on adjacent genes. Furthermore, we found that there is a large positive correlation between *piRNA* density and TE length [*Spearman rank ρ* = 0.684 (sense *piRNA*) and 0.797 (anti-sense *piRNA*), *p <* 10^–16^ for both comparisons]. The positive correlations between H3K9me3 density of a gene and the length of its nearest TE also depend on the distance between genes and TEs [For 0-4hr embryo, *Spearman rank ρ* = 0.151 (short distance between a gene and its nearest TE), 0.120 (intermediate), and 0.083 (long), *p <* 0.01 for all; see S13 Fig for results of all developmental stages], suggesting that the high H3K9me3 of genes near long TEs is probably due to the spread of TE heterochromatin.

Another potential confounding factor comes from the fact that TEs in low recombination regions of the genome are present at higher population frequencies [16,18,48,61]. This observation is generally attributed to lower probability of ectopic recombination [9,10] and/or less effective purifying selection against TEs because of selective interference [62]. Accordingly, we performed logistic regression analyses to investigate the association between genes’ H3K9me3 signature and the population frequencies of nearest TEs while accounting for the effect of recombination rate (see Materials and Methods). We still found that the H3K9me3 density of a gene and the number of developmental stages in which a gene is enriched with H3K9me3 are significant negative predictors for whether the nearest TE is observed in the North American population or not (S11 Table).

## Discussion

Selection removes deleterious TE insertions from the population and plays an important role in the containment of TEs. TE-induced physical disruptions of the genetic elements, such as insertions into functional elements or ectopic recombination between nonhomologous TE insertions, have been viewed as the primary source of the negative fitness impacts of TEs. Empirical investigation and theoretical discussion of TEs’ deleterious impact via epigenetic mechanisms has been limited [22]. In this study, we hypothesized that the *piRNA-*mediated epigenetic silencing of TEs perturbs transcription of adjacent genes and shapes the population dynamics of TEs (Fig 1). Using a genomic approach, we discovered an elevated density of repressive chromatin marks, H3K9me3, in sequences and genes up to several kb away from TEs (Fig 2 and Fig 3), which supports an important component of our hypothesis (Fig 1B and 1C). The H3K9me3 density of a gene heavily depends on its neighboring TE content (number and distance) and the strongest associations were observed for genes that are within 2kb from TEs. These genes account for 10.86% of the euchromatic genes, suggesting that the spread of TEs’ heterochromatin could influence the chromatin status and function of an appreciable number of genes in the genome. In accordance with our hypothesis (Fig 1C), the presence of nearby TEs is also associated with reduced gene expression (Table 1 and S5 Table). Our comparisons of with-TE and without-TE alleles of the same gene clearly demonstrated that the transcriptional consequence of TE insertions is not due to other confounding factors and further extend previous observations on the mutational impacts of TEs on gene expression [55]. Importantly, our observations vary with distance between genes and TEs, where larger distance between genes and TEs are associated with weaker effects. This is consistent with an important aspect of our model that the mutagenic effect of TEs is through the spread of heterochromatin from TEs. Future empirical studies investigating the chromatin states of with-TE and without-TE alleles of the same gene will help further distinguish the causal relationship of these observed associations, which could be attributed to either the spread of TE-induced epigenetic silencing or the preferential insertions of TEs near genes with high heterochromatic marks.

Supporting our hypothesis that the epigenetic silencing of TEs can have deleterious fitness impacts (Fig 1D), we found a *negative* association between H3K9me3 density of a gene and the population frequency of its nearest TE. This observation might be confounded by the fact that TEs adjacent to genes enriched with H3K9me3 tend to be longer, which is expected to increase the rate of ectopic recombination. However, ends of chromosomes that are highly heterochromatic are known to have reduced rates of crossing over. If rates of crossing over are also suppressed at TE-induced heterochromatin in the euchromatic regions, TEs with high H3K9me3 enrichment would be less likely to undergo ectopic recombination, and as a result would be removed by selection less frequently. Under this scenario, we would expect an opposite pattern from our observation—a *positive* correlation between H3K9me3 density of a gene and the population frequency of its nearest TE. In addition, TEs adjacent to genes with high H3K9me3 in multiple tissues also have lower population frequencies. This phenomenon is unlikely to be explained by ectopic recombination removing TEs, but is consistent with the prediction of our hypothesis that TEs epigenetically influencing adjacent genes in more developmental stages have larger overall deleterious impacts. Importantly, the preferential insertion of TEs near genes enriched with heterochromatic marks cannot account for either observation, suggesting that our observed associations between a gene’s H3K9me3 enrichment and its neighborhood TE content should be more attributable to the spread of TE-induced heterochromatin.

We observed that the H3K9me3 density of genes is positively correlated with the *piRNA* density of their nearest TEs, suggesting that our observed patterns depend on the *piRNA-*pathway. Even though the heterochromatin establishment by the *piRNA-*pathway occurs primarily in early embryos [63], the *piRNA-*dependent heterochromatin formed at this stage was found to have a lasting effect and significantly influence the chromatin states in adults [32,63]. Indeed, most of our observations are supported by H3K9me3 data from early embryos through pupae. Accordingly, the epigenetic silencing of TEs during early embryonic development, which depends on maternally deposited *piRNAs*, can influence the chromatin states of adjacent genes that are expressed at different developmental stages and have large cumulative mutational impacts.

Interestingly, the strength and statistical significance of our observed associations between genic H3K9me3 and the properties of their adjacent TEs (including distance from, number, and population frequency) varies across developmental stages. Earlier embryonic stages consistently showed the strongest associations, while larval and pupal stages generally showed the weakest or even statistically insignificant patterns. The effect of TE-induced heterochromatin spreading might be suppressed by other mechanisms of heterochromatin regulation differently at different developmental stages. Intriguingly, flies at later developmental stages, which usually showed weaker associations between genic H3K9me3 density and neighboring TE composition, consist of a greater diversity of differentiated tissues and cell types. It will be important in future work to investigate the tissue specificity of the epigenetic impact of TEs. This can elucidate the temporal and spatial variation in TE-induced heterochromatin spreading and will enable precise identification of the functional effects and evolutionary consequences of TE insertions.

It is worth mentioning that even though our observed associations between genic H3K9me3 density and the neighboring TE content are significant, they are not particularly strong. The paucity of strong correlations might be an issue of power, however. TEs are generally scarce around functional elements [19,64,65] and most TEs appear as singletons in natural populations [4,15–19]. The majority of genes included in our analyses have few adjacent TEs and their nearest TEs are absent in the North American population, providing us limited variation to estimate correlations. In addition, other biological processes can also influence the chromatin state of genes. By comparing chromatin states across a single genome (which was the focus of most of our analyses), we were unable to distinguish the epigenetic effects of TEs from those of other biological processes. Investigation of variation in chromatin states between “with TE” and “without TE” alleles, which presumably only differ with respect to their neighboring TE composition, can help further address the relative importance of the epigenetic effects of TEs on the chromatin states of genes and the evolutionary dynamics of TEs.

It was previously reported that the epigenetic silencing of TEs via methylation could influence the expression of adjacent genes in *Arabidopsis thaliana* [22]. Epigenetically silenced (methylated) TEs, but not unmethylated TEs, were found to be associated with lower expression of nearby genes and are present in lower population frequencies. Even though DNA methylation is rare in the *D*. *melanogaster* genome and definite evidence for methylated TEs is still missing [66,67], we observed a similar epigenetic impact of TEs on the expression of neighboring genes with a different mechanism (histone modifications). We provided complementary observations by investigating the associations between the heterochromatic mark enrichment of *genes* and their neighborhood TE composition. Importantly, combining our observed elevated H3K9me3 density surrounding TEs and the PEV phenomenon that has been known for decades ([34], reviewed in [35–37]), we are able to provide a mechanistic explanation for the observed deleterious impacts of TE’s epigenetic silencing.

There are several properties that make the *piRNA-*mediated epigenetic silencing of TEs an especially attractive mechanism for containment of TE copy number in natural populations. The generation of *piRNAs* only depends on the transcription of TE sequences and is thus general to virtually all classes and families of TEs [20]. Importantly, unlike other small RNAs, *piRNAs* are generated and amplified through a feed-forward ping-pong cycle (*i*.*e*. a positive feedback loop) [23,24]. The TE transcripts are targeted by anti-sense *piRNAs* and processed into sense-*piRNAs*, which are involved in generating additional antisense *piRNAs*. Despite previous suggestions that antisense *piRNAs* are mostly generated from *piRNA* clusters in heterochromatic regions [24], a recent functional study [49] and statistical analysis [59] both showed that euchromatic TE copy number is a major determinant of *piRNA* amount. It is expected that the probability that the DNA sequence of a TE is targeted by *piRNAs* increases with *piRNA* amount and, accordingly, the number of TEs that are epigenetically silenced will depend both on the amount of *piRNAs* (which is positively correlated with TE copy number of a family [59]) and TE copy number. Consequently, the number of epigenetically silenced TEs and the resulting mutational impacts due to TE-heterochromatin spreading might depend quadratically on TE copy number. This can potentially provide the required synergistic epistasis of TEs’ deleterious impacts for stable containment of TE copy number [13].

Our study demonstrates that the spread of *piRNA-*mediated heterochromatin of TEs is another important, though previously unexplored, mechanism leading to removal of TEs. Given *piRNA’s* wide phylogenetic distribution in animals [68], we expect this selective mechanism against TEs also plays an important role in the containment of TEs in other organisms. Further investigations of the relative roles of different selective mechanisms in the containment of TEs, particularly the potential interference between our proposed TE-induced indirect epigenetic effects and the widely empirically supported ectopic recombination between TEs, will help piece together our picture of TE dynamics in natural populations.

## Materials and Methods

### Genome annotations

Our analyses used *D*. *melanogaster* reference genome annotation 5.21 for genes, TEs, and other functional sequences. We only included genes and TEs that are in the euchromatic regions of the genome, using the heterochromatin-euchromatin boundaries defined in [69]. Genes and TEs on the 4^th^ chromosome were excluded from the analyses as well.

### Processing H3K9me3 ChIP-seq data

We used H3K9me3 ChIP-seq data generated by modEncode [40], which used samples from nine developmental stages of the reference *D*. *melanogaster* strain (0-4hr, 4-8hr, 8-12hr, 12-16hr, 16-20hr, and 20-24hr embryos, and L1 larvae, L2 larvae, and pupae). We used the modEncode processed wiggle files, which are background subtracted H3K9me3 read density (normalized read counts of uniquely mapped reads of H3K9me3 experiments subtracting the re-scaled read counts of uniquely mapped reads of the input experiments, [70]). Windows that have negative background-subtracted read density (no enrichment of H3K9me3) were assigned to zero.

### The effect of TEs on the H3K9me3 density of adjacent intergenic sequences

The average H3K9me3 read density of the upstream/downstream 10kb sequences of TEs was estimated in 1kb nonoverlapping windows. We excluded 10kb sequences that overlap with any annotations other than “intergenic” and analyzed the right side and left side of the sequences separately. To generate a null expectation for the H3K9me3 density decay near TEs, we randomly chose sequences that have the same size and are on the same chromosome as TEs included in the analyses. To account for the observed large-scale variation in histone modifications across *D*. *melanogaster* genome [39,40], we divided each chromosomal arm into 4Mb bins (5–7 bins per chromosome) and randomly selected “TE-size” segments within the same bins as the reference TEs. The number of randomly chosen sequences is the same as the number of TEs included in the analysis. For each randomly selected segment, we estimated the H3K9me3 density of adjacent sequences using the same methods as for TEs. This procedure was repeated 1,000 times.

### The effect of TEs on the H3K9me3 density of adjacent genes

The H3K9me3 density of genes was estimated as weighted averages across all exons of the longest isoforms. We excluded genes that have TEs inside their coding or noncoding exons. We further removed genes whose 10kb upstream/downstream sequences overlaps with the euchromatin-heterochromatin boundaries, because of how we categorized genes according to their neighboring TE content (see below). Genes are categorized into nonoverlapping groups according to their distance from the nearest TE (having TEs in introns, in 1kb, in 1-2kb, in 2-5kb, and in 5-10kb upstream/downstream of the gene, or have no TEs 10kb upstream/downstream of the gene). These genes account for 3.66% (in gene), 4.65% (in 1kb), 2.55% (1-2kb), 6.83% (2-5kb), and 9.26% (5-10kb) of the analyzed euchromatic genes (12,204 genes). We also counted the number of TEs that are inside introns, less than 1kb, 1-2kb, 2-5kb, and 5-10kb away from each gene. For TEs that span over multiple windows, we classified them to windows that are closest to genes. For analyses considering the properties of nearest TEs, genes that have more than one TEs of equal distance were excluded, resulting in 3,193 gene-TE pairs. Gene-TE pairs were further categorized into three equal-size bins according to the distance between them (short: 0-1451bp, intermediate: 1452-5184bp, and long: > 5184bp). Recombination rate were interpolated for the mid-point of genes or TEs using [71]. Gene density was estimated as the number of genes in a 100kb window centered on the focused gene. S12 Table includes estimated H3K9me3 density, TE states, and other genic attributes of analyzed genes.

### Estimation of *piRNA* density

The ovarian *piRNA* of reference strain [49] were processed and mapped without mismatches (using BWA [72]) to reference genome release 5 following methods in [73]. We estimated the density (average per bp) of *piRNAs* mapped to sense (sense *piRNA*) and antisense (antisense *piRNA*) strands of genes/TEs. For *piRNAs* mapped to multiple genomic locations, each of the *n* mapped positions in the genome is counted as having *1/n* read mapped. Analyses using all mapped *piRNAs* (uniquely mapped and multiply mapped) or only uniquely mapped *piRNAs* gave consistent results. We presented the results based on all mapped *piRNAs* because this represents the full probability that a TE is targeted by the *piRNA-*pathway.

### Testing the expressional impacts of TEs

To investigate the impact of H3K9me3 on gene expression, we used modEncode developmental stage expression data that were generated also using reference *D*. *melanogaster* strain [74]. The normalized and standardized expression level (RPKM) was downloaded from FlyBase. No (zero) expression of genes could be due to the presence of nearby TE insertions or simply the absence of expression at a particular developmental stage. Because we could not distinguish these alternatives, we excluded genes that have zero expression in the analysis of the particular developmental stage.

There are 21 strains of a North American population that have both microarray-based gene expression data [54] and TE calls [19]. We used these strains to investigate the expressional impacts of TEs within population. We downloaded processed gene expression data from the supplementary data of [54]. To avoid the systematic difference in expression level across microarray experiments and/or individuals, we used expressional rank (from highest to lowest) within each samples. In [19], for each strain, each TE insertion site was annotated as “present” (with TE), “absent” (no TE), or “no call” (missing data due to low sequencing coverage). For each gene, the “with TE” alleles are those that have one or more TE insertion sites called as “present” within introns, in 1kb, 2kb, 5kb, or 10kb windows from the gene. An allele is categorized as “without TE” if all the known TE insertion sites near it were called as “absent”. An allele is treated as missing data when any of the known TE insertion sites near it was called as “no call” and none of them were called as “present” (see S13 Table for TE states of all alleles). For genes that have at least two “with TE” and at least two “without TE” alleles, we calculated the difference in mean expression rank between “with TE” alleles and “without TE” alleles. For each gene, we used all possible permutations with respect to TE labels to find the null distribution of rank differences between “with TE” and “without TE” alleles (“with TE” alleles minus “without TE” alleles). Positive differences suggest that the “with TE” alleles have larger expressional rank, or lower expression, than “without TE” alleles. One tail *p-values* were calculated as the proportion of permuted combinations that have differences *greater than or equal to* the observed differences. To ensure that a gene could potentially have a significant (*p* < 0.05) *p-value*, we further restricted our analysis to genes that have more than 20 possible permuted combinations. We also randomly chose the same number of genes that have no TE alleles (“without TE genes”) as the number of “genes with TE alleles”. The alleles of these “without TE genes” were randomly partitioned into two groups accordingly to the observed allele frequencies for “genes with TE alleles”, and we used these “without TE genes” to assess the false positive rate of above procedures. This process was repeated 100 times for each window size and separately for each sex. We found that the false positive rate of our approach is slightly smaller than the expected 5% (S14 Table).

### Variation in selective pressure on TEs

We used the same TE polymorphism data of the North American population [19] to investigate the evolutionary impacts of heterochromatin spreads from TEs. It is worth noting that this part of the analyses included all 131 genomes whose TEs has been annotated in the North American population [19], compared to 21 in the above expressional impact analysis. Only TEs observed in the reference genome were included in our analysis. For TEs that are only observed in the reference genome, their population frequencies in the North American population is zero.

We performed two-way Analysis of Variance analysis (ANOVA) to test whether the nearest TE is observed in the North American population (binary variable), the family of nearest TE (categorical variable, 52 TE families), and the interaction between these two variables contribute to the variation in H3K9me3 density of genes (model: H3K9me3 density ~ observed/not + TE family + observed/not * TE family). The H3K9me3 density of genes has an overall exponential distribution and a large number of genes with zero H3K9me3 density, which made us unable to identify appropriate transformation for the response variable (H3K9me3 density). Accordingly, we restricted our analysis to genes that have positive H3K9me3 density and log-transformed the H3K9me3 density of genes. We also performed mixed linear model analysis to investigate the influence of TE frequency on genic H3K9me3 while treating the influence of TE family as random. Mixed linear model regression was performed using R package nlme version 3.1.

We performed logistic regressions, using whether a *reference TE* is observed (one) or not (zero) in the North American population as response variable and the H3K9me3 status of its nearest gene and TE’s local recombination rate as predictors. We first performed regression analysis that included only one predictor variable at a time to determine the regression model (linear, quadratic, or logarithmic). We then included all predictor variables and performed backward model selection based on AIC to determine the regression model. Full regression models used are:

$$logit\;p\sim \text{H}3\text{K}9\text{me}3\;\text{density}+{\left(\text{H}3\text{K}9\text{me}3\;\text{density}\right)}^{2}+\text{recombination rate}+{\left(\text{recombination rate}\right)}^{2},$$

$$logit\;p\sim \text{no}.\;\text{dev}.\;\text{stages}+{\left(\text{no}.\;\text{dev}.\;\text{stages}\right)}^{2}+\text{recombination rate}+{\left(\text{recombination rate}\right)}^{2}{,}^{}$$

where *logit p* is the log odds of whether a reference TE is observed in the North American population (one) or not (zero) and “no. dev. stages” is the number of developmental stages a gene has top 10% H3K9me3 density genome-wide. All statistical analyses were performed using R.

## Supporting Information

S1 Fig The decay of H3K9me3 density of intergenic sequences adjacent to TEs (median). The observed median H3K9me3 densities for windows adjacent to TEs are higher than those adjacent to randomly chosen TE-size sequences (gray lines, 1,000 sets of randomly chosen TE-size sequences) for most developmental stages, particularly for windows that are closest to TEs.
(PDF)

S2 Fig The decay of H3K9me3 density of intergenic sequences adjacent to TEs (mean). The observed mean H3K9me3 densities for windows adjacent to TEs are higher than those adjacent to randomly chosen TE-size sequences (gray lines, 1,000 sets of randomly chosen TE-size sequences), particularly for windows that are closest to TEs.
(PDF)

S3 Fig The H3K9me3 density of genes that are of different distance from TEs. Boxplots for the H3K9me3 density of genes that are of different distance from TEs are shown for all developmental stages. Genes that are farther away from TEs have lower H3K9me3 density. Dashed lines represent the median of the H3K9me3 of genes that do not have TEs within 10kb upstream and downstream. H3K9me3 densities of genes that have TEs within 10kb are compared to those of genes without TEs in 10kb, using *Mann-Whitney U test*. Notations for *p-values* are * (*p* < 0.05), ** (*p* < 0.01), and *** (*p* < 0.001)
(PDF)

S4 Fig The H3K9me3 density of genes that are of different distance from TEs, excluding genes that have high H3K9me3 in Oregon-R strain. Boxplots for the H3K9me3 density of genes that are of different distance from TEs are shown for all developmental stages. Genes that have high H3K9me3 in embryonic or larval tissues of Oregon-R strain are excluded from the analyses and consistent patterns were observed—genes that are farther away from TEs have lower H3K9me3 density. Dashed lines represent the median of the H3K9me3 of genes that do not have TEs within 10kb upstream and downstream. H3K9me3 densities of genes that have TEs within 10kb are compared to those of genes without TEs in 10kb, using *Mann-Whitney U test*. Notations for *p-values* are * (*p* < 0.05), ** (*p* < 0.01), and *** (*p* < 0.001)
(PDF)

S5 Fig The H3K9me3 density of genes that are of different distance from TEs, excluding genes that are in genomic regions annotated as state 7 by modEncode. Boxplots for the H3K9me3 density of genes that are of different distance from TEs are shown for all developmental stages. Genes that have high H3K9me2/3 in either S2 or BG3 cells (state 7) are excluded from the analyses and consistent patterns were observed—genes that are farther away from TEs have lower H3K9me3 density. Dashed lines represent the median of the H3K9me3 of genes that do not have TEs within 10kb upstream and downstream. H3K9me3 densities of genes that have TEs within 10kb are compared to those of genes without TEs in 10kb, using *Mann-Whitney U test*. Notations for *p-values* are * (*p* < 0.05), ** (*p* < 0.01), and *** (*p* < 0.001)
(PDF)

S6 Fig The H3K9me3 density of genes that are of different distance from TEs, excluding genes that are in genomic regions annotated as state 7 or 8 by modEncode. Boxplots for the H3K9me3 density of genes that are of different distance from TEs are shown for all developmental stages. Genes that have high H3K9me2/3 (state 7) or moderate H3K9me2/3 (state8) in either S2 or BG3 cells are excluded from the analyses and consistent patterns were observed—genes that are farther away from TEs have lower H3K9me3 density. Dashed lines represent the median of the H3K9me3 of genes that do not have TEs within 10kb upstream and downstream. H3K9me3 densities of genes that have TEs within 10kb are compared to those of genes without TEs in 10kb, using *Mann-Whitney U test*. Notations for *p-values* are * (*p* < 0.05), ** (*p* < 0.01), and *** (*p* < 0.001)
(PDF)

S7 Fig The *Spearman rank* correlation coefficients between H3K9me3 density of genes and the number of adjacent TEs, excluding genes that have high H3K9me3 in the Oregon-R strain. Genes that have high H3K9me3 density in the Oregon-R strain are removed from the analyses. The correlations are stronger for windows that are closer to the gene. Notations for *p-values* are * (*p* < 0.05), ** (*p* < 0.01), and *** (*p* < 0.001)
(PDF)

S8 Fig The *Spearman rank* correlation coefficients between H3K9me3 density of genes and the number of adjacent TEs, excluding genes that are in genomic regions annotated as state 7 by modEncode. Genes that have high H3K9me2/3 (state 7) in either S2 or BG3 cells are excluded from the analyses and consistent patterns were observed. Notations for *p-values* are * (*p* < 0.05), ** (*p* < 0.01), and *** (*p* < 0.001)
(PDF)

S9 Fig The *Spearman rank* correlation coefficients between H3K9me3 density of genes and the number of adjacent TEs, excluding genes that are in genomic regions annotated as state 7 or 8 by modEncode. Genes that have high H3K9me2/3 (state 7) or moderate H3K9me2/3 (state 8) in either S2 or BG3 cells are excluded from the analyses and consistent patterns were observed. Notations for *p-values* are * (*p* < 0.05), ** (*p* < 0.01), and *** (*p* < 0.001).
(PDF)

S10 Fig The *partial Spearman rank* correlation coefficients between H3K9me3 density of genes and the number of adjacent TEs, controlling for local gene density. Partial correlation analyses were performed to account for the correlations between H3K9me3 density of genes and their local gene density. The analyses still found significant correlations between H3K9me3 density of a gene. In addition, the number of adjacent TEs within a specific window and the correlations decrease as the distance between the gene and the designed window increases. Notations for *p-values* are * (< 0.05), ** (< 0.01), *** (< 0.001).
(PDF)

S11 Fig The *partial Spearman rank* correlation coefficients between H3K9me3 density of genes and the number of adjacent TEs, controlling for local recombination rate. Partial correlation analyses were performed to account for the correlations between H3K9me3 density of genes and their local recombination rate. The analyses still found significant correlations between H3K9me3 density of a gene and the number of adjacent TEs within a specific window. In addition, the correlations decrease as the distance between the gene and the designed window increases. Notations for *p-values* are * (< 0.05), ** (< 0.01), *** (< 0.001).
(PDF)

S12 Fig Relationships between genic H3K9me3 at embryo 0–4 hrs and *piRNAs* targeting its nearest TE. (A) Gene-TE pairs were classified into three equal–size groups according to their distance from the nearest TEs (short, intermediate, and long). TEs of these gene-TE pairs were categorized into five equal bins according to their *piRNA* density (1–5, from lowest to highest *piRNA* density). Figures are boxplots for genes whose nearest TEs are of different *piRNA* bins. Genes whose nearest TEs are targeted by higher *piRNA* density have higher H3K9me3 density and this trend is more pronounced for genes that are closer to TEs. The same data presented as scatter plots are shown in (B).
(PDF)

S13 Fig The *Spearman rank* correlation coefficients between H3K9me3 density of a gene and the length of its nearest TE. The correlations decrease as the distances between the gene and the TE increase (represented with different color bars). Notations for *p-values* are * (< 0.05), ** (< 0.01), *** (< 0.001).
(PDF)

S1 Table Decay of H3K9me3 density of intergenic sequences adjacent to TEs. The H3K9me3 density of 1kb windows that are of different distance from TEs were compared to those of window that are 9-10kb away from TEs using *Mann-Whitney U test*.
(PDF)

S2 Table *Spearman rank* correlation coefficients between a gene’s H3K9me3 density and distance from the nearest TE. (PDF)

S3 Table Comparisons of genic and TE’s *piRNA* density. A small fraction of *piRNAs* was found mapped to genes. The *piRNA* densities of genes are significantly lower than the *piRNA* densities of TEs. Correlations between genic H3K9me3 density and *piRNA* density of genes/nearest TEs were estimated using *Spearman Rank correlation* test. Unlike the correlations between a gene’s H3K9me3 density and the *piRNA* density of its nearest gene, we did not observe positive correlations between a gene’s H3K9me3 density and its *piRNA* density.
(PDF)

S4 Table *Spearman rank* correlation coefficient between H3K9me3 density of a gene and its expression level for different developmental stages. The correlations were performed for all euchromatic genes included in the analyses (all genes) as well as for genes that have TEs within 10kb (genes with TE in 10kb).
(PDF)

S5 Table Comparisons of expression level between genes with and without adjacent TEs. Genes are categorized according to their distance from the nearest TE (up to 10kb). The expression levels of each category genes that have TEs nearby are compared to expression levels of genes without TEs nearby using *Mann-Whitney U test*.
(PDF)

S6 Table Permutation results for testing differential expression between “with TE” and “without TE” alleles in female. (TXT)

S7 Table Permutation results for testing differential expression between “with TE” and “without TE” alleles in male. (TXT)

S8 Table Number of genes that showed differential expression between “with TE” and “without TE” alleles in both female and male. (PDF)

S9 Table *Spearman rank* correlation coefficients between a gene’s H3K9me3 density and the population frequency of its nearest TE. (PDF)

S10 Table ANOVA result for testing the association between genic H3K9me3 density and nearest TEs’ population frequency. TEs of gene-TE pairs were classified into those that are observed (Observed) and not observed (Not observed) in a North American *D*. *melanogaster* population. We performed two-way ANOVA to test whether, when accounting for the effect of TE families, the H3K9me3 density of genes varies according to the population frequencies of their nearest TEs (model: H3K9me3 density ~ observed/not + TE family + observed/not * TE family). Our analysis showed that TE frequency has a significant effect on variation of genic H3K9me3 density at six developmental stages. We used two ways to infer the directionality of the influence of TE frequency on genic H3K9me3 density while taking into account the influence of TE families. According to the results of ANOVA, the interaction terms are *not* significant for all except one developmental stage at which TE frequency has a significant effect on H3K9me3 density (embryo 4–8 hr, 8–12 hr, 16–20 hr, 20–24 hr, and L2 larvae), suggesting that the directionality of the differences in H3K9me3 density (*i*.*e*. “Not observed” genes have higher H3K9me3 density) is consistent across TE families. Second, we performed linear regression to examine the signs for the coefficients of TE frequency (categorical “observed/not” was changed to numerical “TE frequency”). For developmental stages at which the influence of TE frequency on genic H3K9me3 density is significant in the ANOVA analysis, the regression coefficients of TE frequency have negative signs except for one developmental stage. It is worth noting that, because of the large number of TE families (50 families were included in this analysis), few terms are significant in the linear regression analysis due to the reduction in degrees of freedom. Alternatively, we treated the effect of TE family on genic H3K9me3 density as random and performed mixed linear model analysis. The coefficients of TE frequency are significant for four developmental stages and they all have negative signs. Our analyses suggest that, while taking into account the influence of TE families, there is still a negative association between genic H3K9me3 density and the population frequency of nearest TEs.
(PDF)

S11 Table Regression analyses for testing the association between genic H3K9me3 and nearest TEs’ population frequency. Terms that are not selected in the backward AIC selection analysis are not included in the final regression model (denoted as “NA”). See Materials and Methods for regression models used.
(PDF)

S12 Table H3K9me3 density, TE status, and other genic attributes for euchromatic genes analyzed. (TXT)

S13 Table TE states for the 21 strains of the North American population. (TXT)

S14 Table False positive rate for the permutation procedure. This table summarizes, for 100 randomly selected sets of genes that have no TEs within 10kb, the proportion of genes with significant (one-tailed *p-value* < 0.05) differential expression between two randomly partitioned groups of alleles (see Materials and Methods). This was used to assess the false positive rate for the permutation procedure.
(PDF)
