# Supplementary figures and images for: The Role of piRNA-Mediated Epigenetic Silencing in the Population Dynamics of Transposable Elements in Drosophila melanogaster
Source: PLoS Genet. 2015 Jun 4;11(6):e1005269. doi: 10.1371/journal.pgen.1005269 (PMC4456100; doi:10.1371/journal.pgen.1005269)

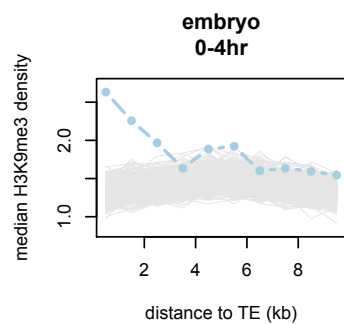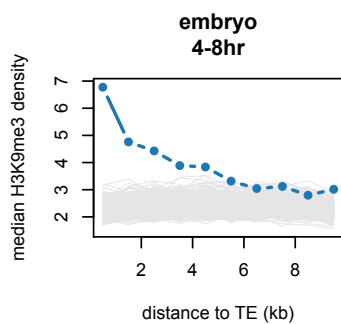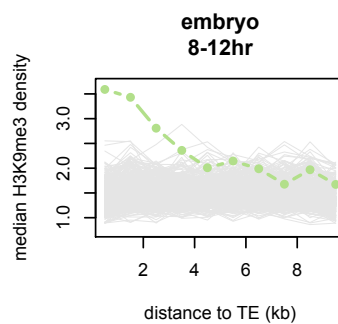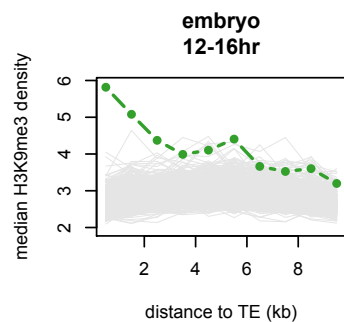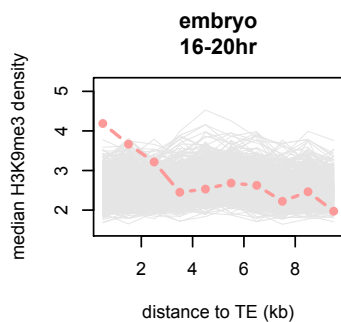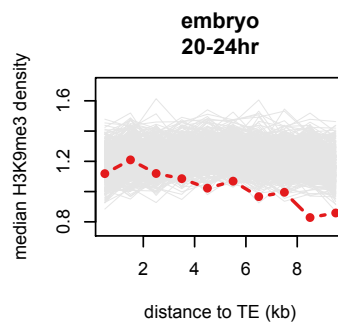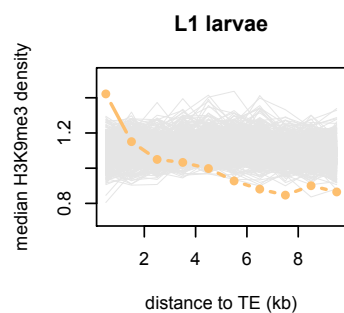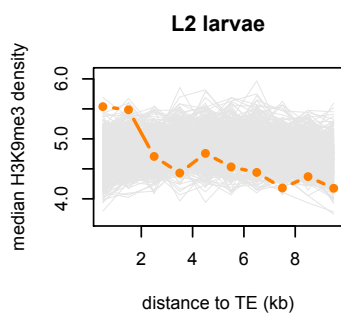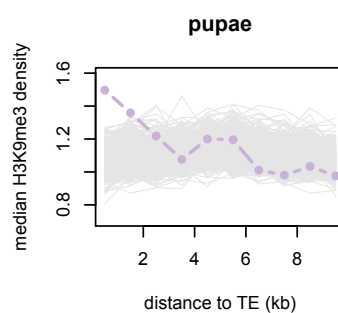

Supplement: S1 Fig — The observed median H3K9me3 densities for windows adjacent to TEs are higher than those adjacent to randomly chosen TE-size sequences (gray lines, 1,000 sets of randomly chosen TE-size sequences) for most developmental stages, particularly for windows that are closest to TEs. (PDF) [file pgen.1005269.s001.pdf]

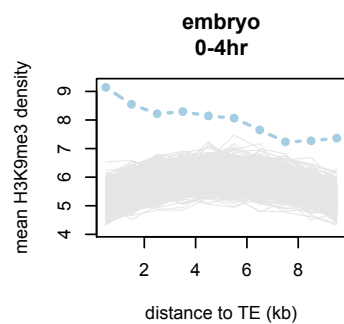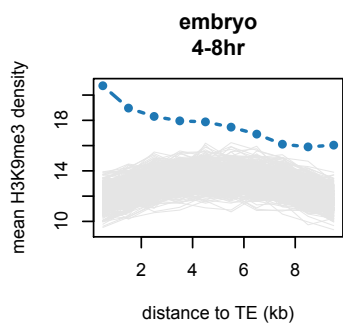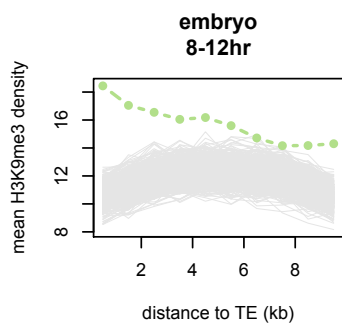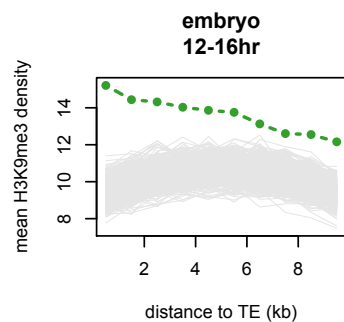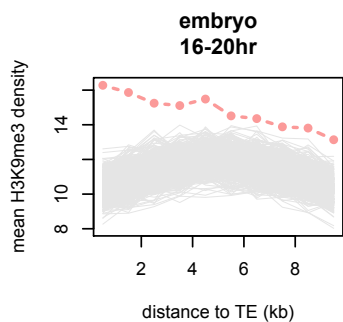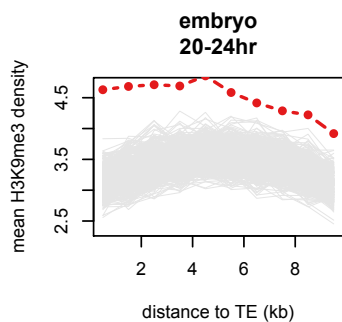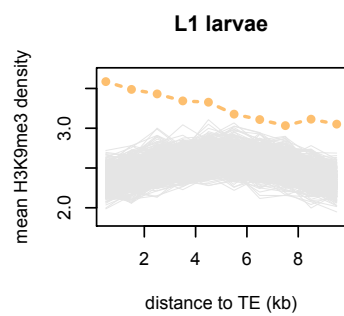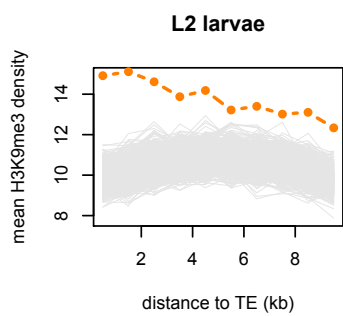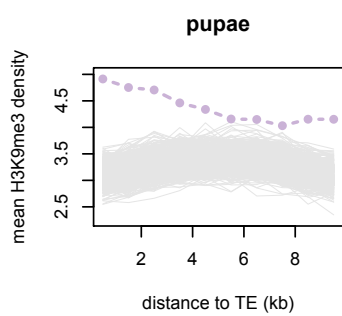

Supplement: S2 Fig — The observed mean H3K9me3 densities for windows adjacent to TEs are higher than those adjacent to randomly chosen TE-size sequences (gray lines, 1,000 sets of randomly chosen TE-size sequences), particularly for windows that are closest to TEs. (PDF) [file pgen.1005269.s002.pdf]

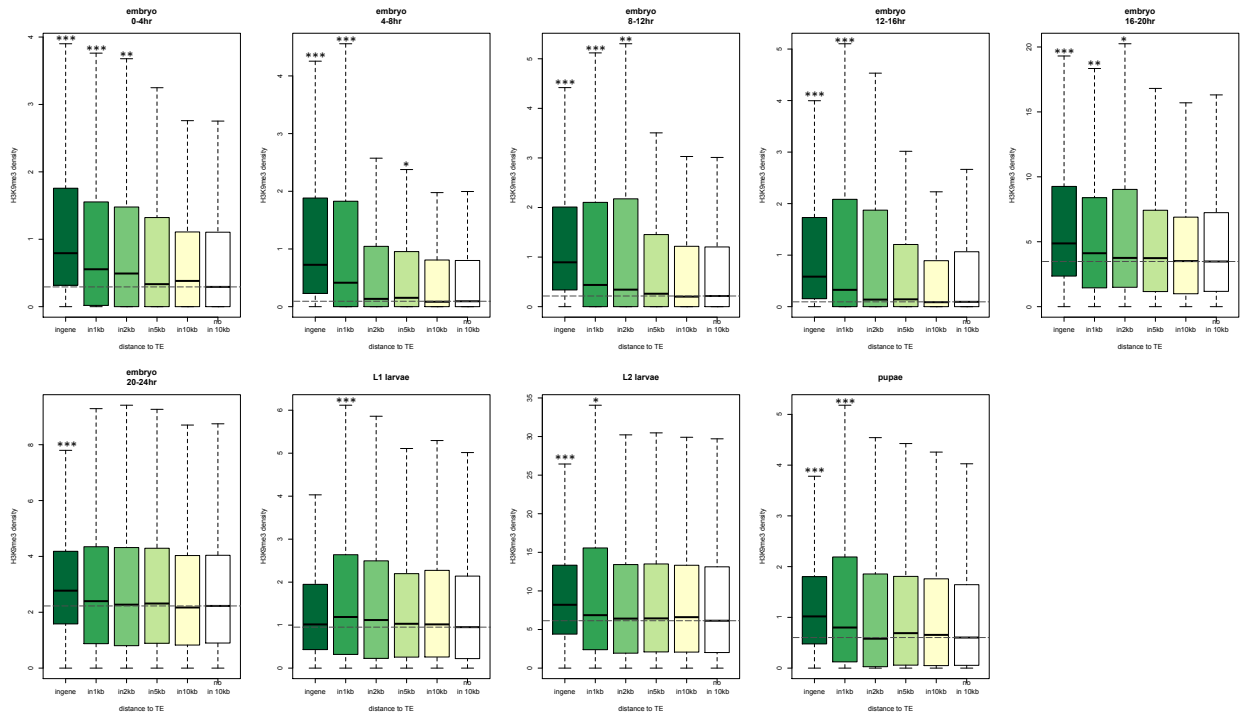

Supplement: S3 Fig — Boxplots for the H3K9me3 density of genes that are of different distance from TEs are shown for all developmental stages. Genes that are farther away from TEs have lower H3K9me3 density. Dashed lines represent the median of the H3K9me3 of genes that do not have TEs within 10kb upstream and downstream. H3K9me3 densities of genes that have TEs within 10kb are compared to those of genes without TEs in 10kb, using Mann-Whitney U test. Notations for p-values are * (p < 0.05), ** (p < 0.01), and *** (p < 0.001) (PDF) [file pgen.1005269.s003.pdf]

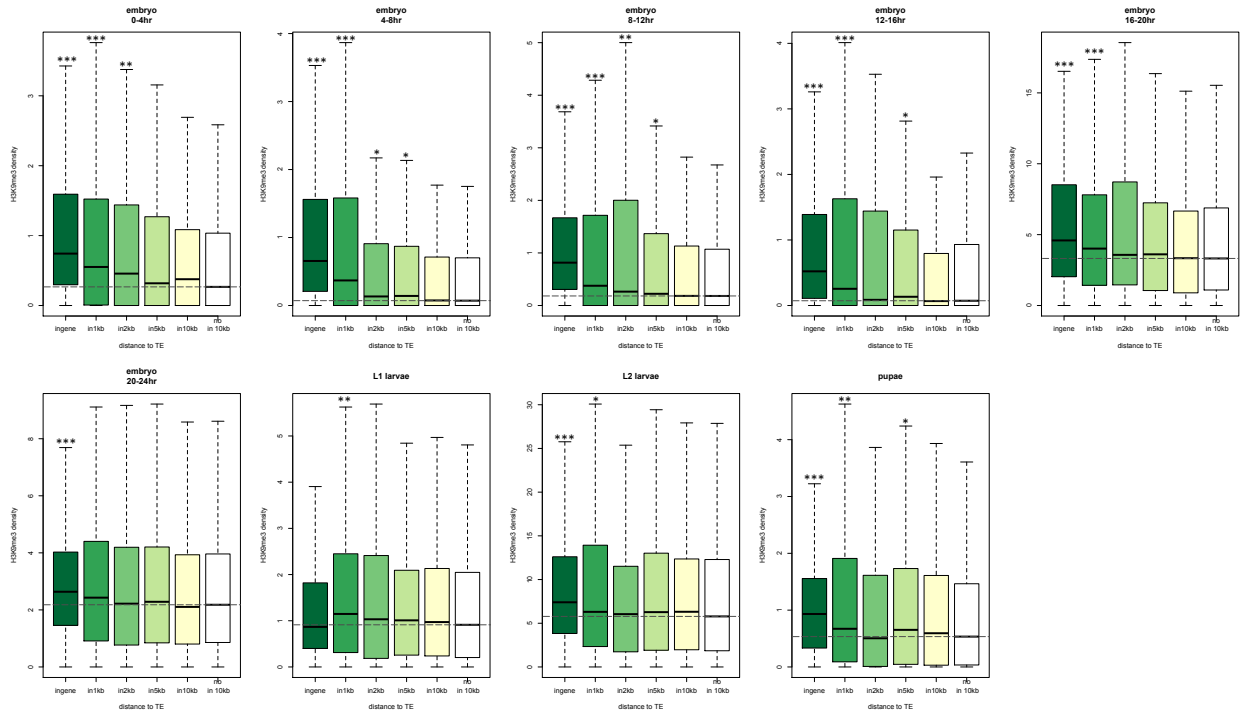

Supplement: S4 Fig — Boxplots for the H3K9me3 density of genes that are of different distance from TEs are shown for all developmental stages. Genes that have high H3K9me3 in embryonic or larval tissues of Oregon-R strain are excluded from the analyses and consistent patterns were observed—genes that are farther away from TEs have lower H3K9me3 density. Dashed lines represent the median of the H3K9me3 of genes that do not have TEs within 10kb upstream and downstream. H3K9me3 densities of genes that have TEs within 10kb are compared to those of genes without TEs in 10kb, using Mann-Whitney U test. Notations for p-values are * (p < 0.05), ** (p < 0.01), and *** (p < 0.001) (PDF) [file pgen.1005269.s004.pdf]

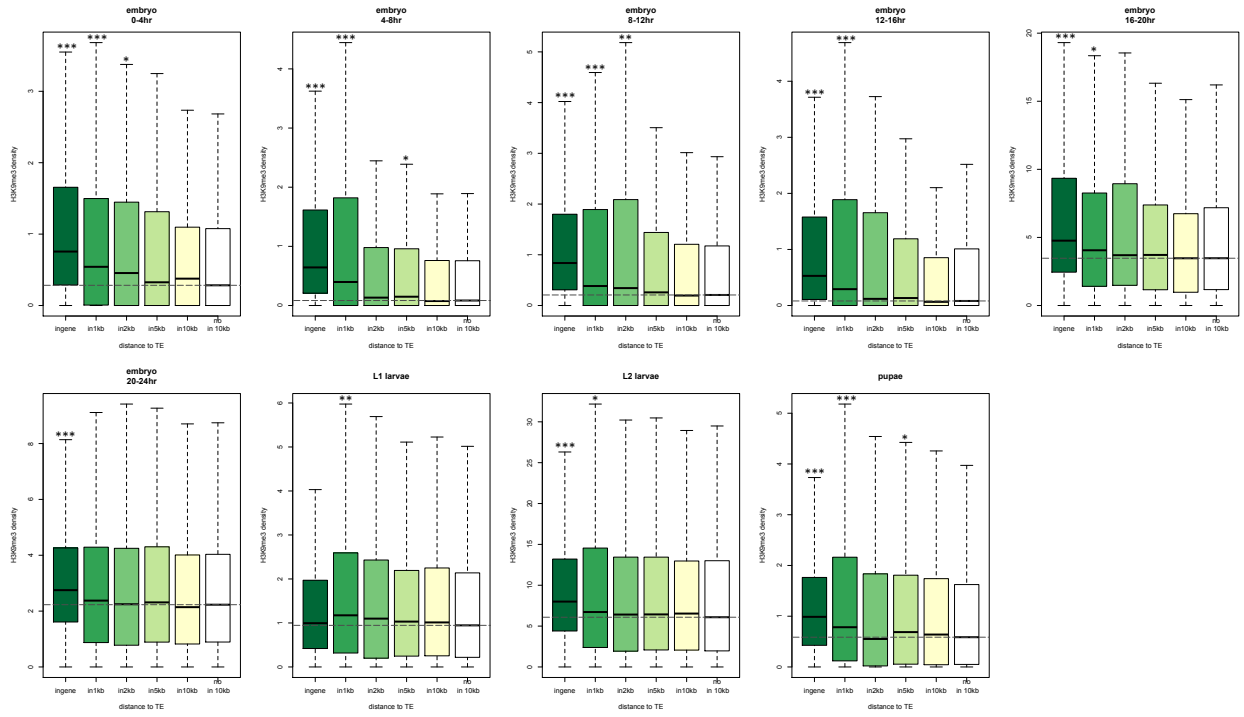

Supplement: S5 Fig — Boxplots for the H3K9me3 density of genes that are of different distance from TEs are shown for all developmental stages. Genes that have high H3K9me2/3 in either S2 or BG3 cells (state 7) are excluded from the analyses and consistent patterns were observed—genes that are farther away from TEs have lower H3K9me3 density. Dashed lines represent the median of the H3K9me3 of genes that do not have TEs within 10kb upstream and downstream. H3K9me3 densities of genes that have TEs within 10kb are compared to those of genes without TEs in 10kb, using Mann-Whitney U test. Notations for p-values are * (p < 0.05), ** (p < 0.01), and *** (p < 0.001) (PDF) [file pgen.1005269.s005.pdf]

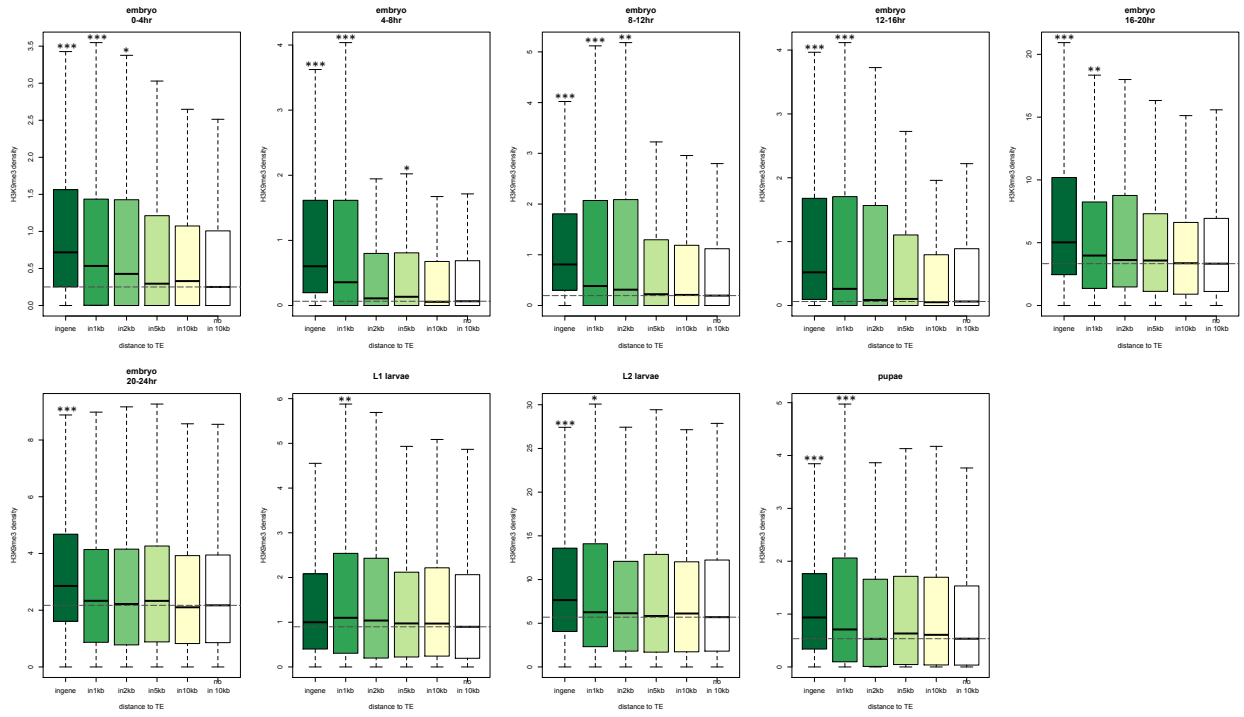

Supplement: S6 Fig — Boxplots for the H3K9me3 density of genes that are of different distance from TEs are shown for all developmental stages. Genes that have high H3K9me2/3 (state 7) or moderate H3K9me2/3 (state8) in either S2 or BG3 cells are excluded from the analyses and consistent patterns were observed—genes that are farther away from TEs have lower H3K9me3 density. Dashed lines represent the median of the H3K9me3 of genes that do not have TEs within 10kb upstream and downstream. H3K9me3 densities of genes that have TEs within 10kb are compared to those of genes without TEs in 10kb, using Mann-Whitney U test. Notations for p-values are * (p < 0.05), ** (p < 0.01), and *** (p < 0.001) (PDF) [file pgen.1005269.s006.pdf]

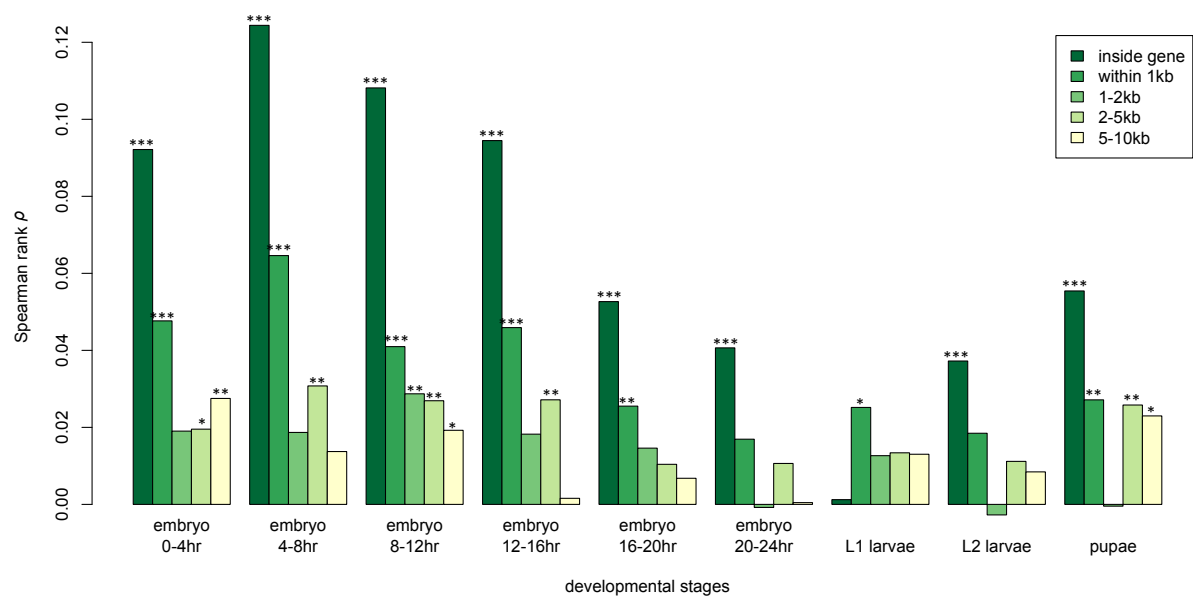

Supplement: S7 Fig — Genes that have high H3K9me3 density in the Oregon-R strain are removed from the analyses. The correlations are stronger for windows that are closer to the gene. Notations for p-values are * (p < 0.05), ** (p < 0.01), and *** (p < 0.001) (PDF) [file pgen.1005269.s007.pdf]

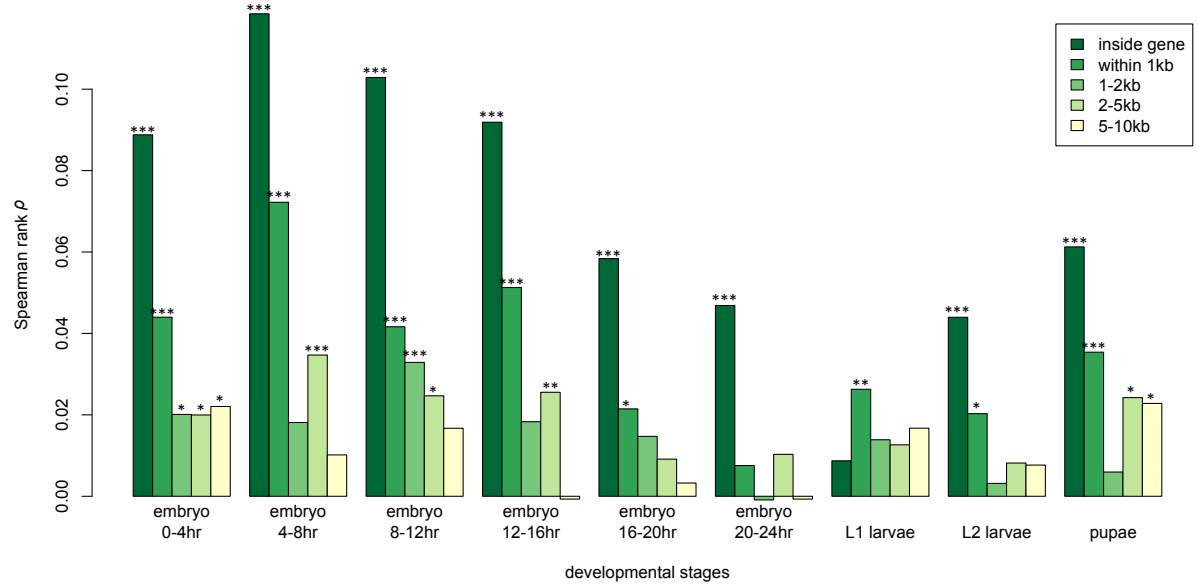

Supplement: S8 Fig — Genes that have high H3K9me2/3 (state 7) in either S2 or BG3 cells are excluded from the analyses and consistent patterns were observed. Notations for p-values are * (p < 0.05), ** (p < 0.01), and *** (p < 0.001) (PDF) [file pgen.1005269.s008.pdf]

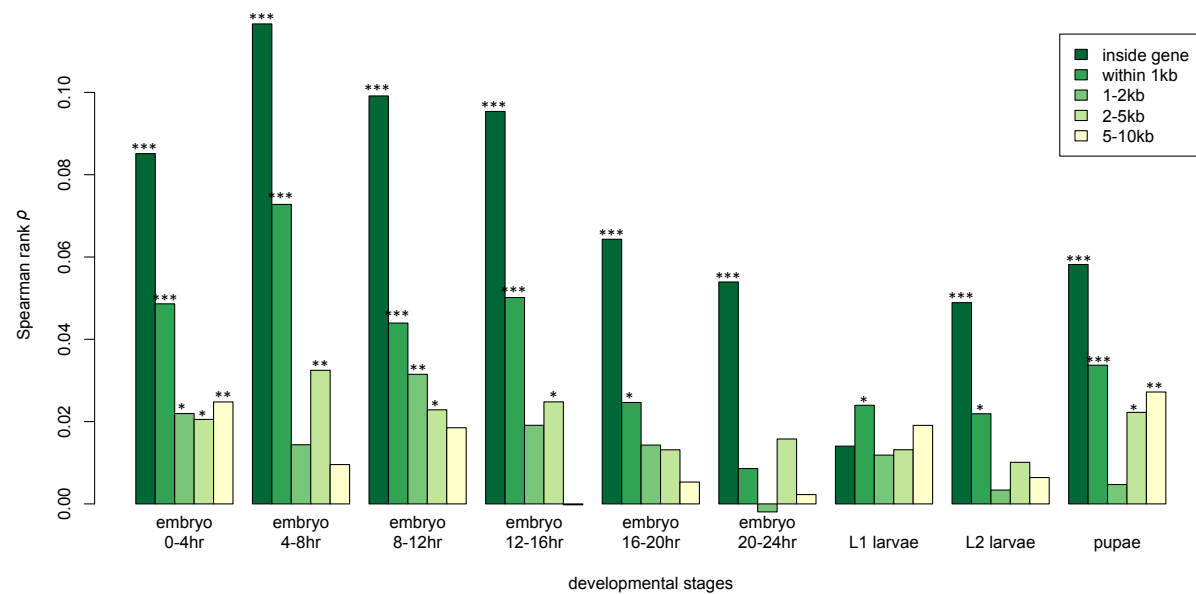

Supplement: S9 Fig — Genes that have high H3K9me2/3 (state 7) or moderate H3K9me2/3 (state 8) in either S2 or BG3 cells are excluded from the analyses and consistent patterns were observed. Notations for p-values are * (p < 0.05), ** (p < 0.01), and *** (p < 0.001). (PDF) [file pgen.1005269.s009.pdf]

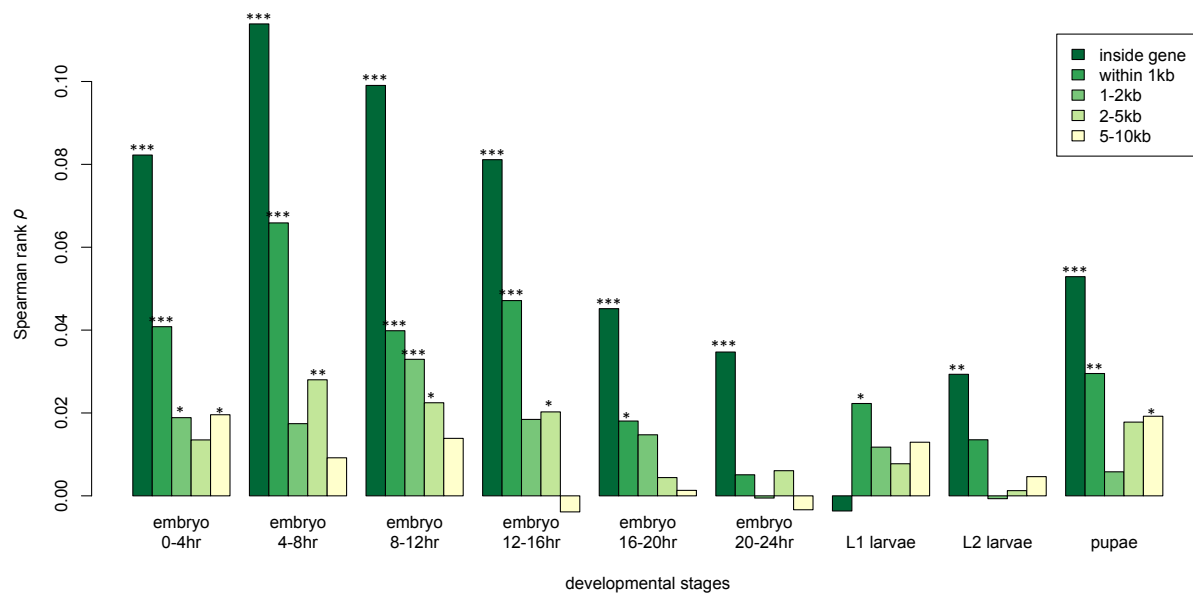

Supplement: S10 Fig — Partial correlation analyses were performed to account for the correlations between H3K9me3 density of genes and their local gene density. The analyses still found significant correlations between H3K9me3 density of a gene. In addition, the number of adjacent TEs within a specific window and the correlations decrease as the distance between the gene and the designed window increases. Notations for p-values are * (< 0.05), ** (< 0.01), *** (< 0.001). (PDF) [file pgen.1005269.s010.pdf]

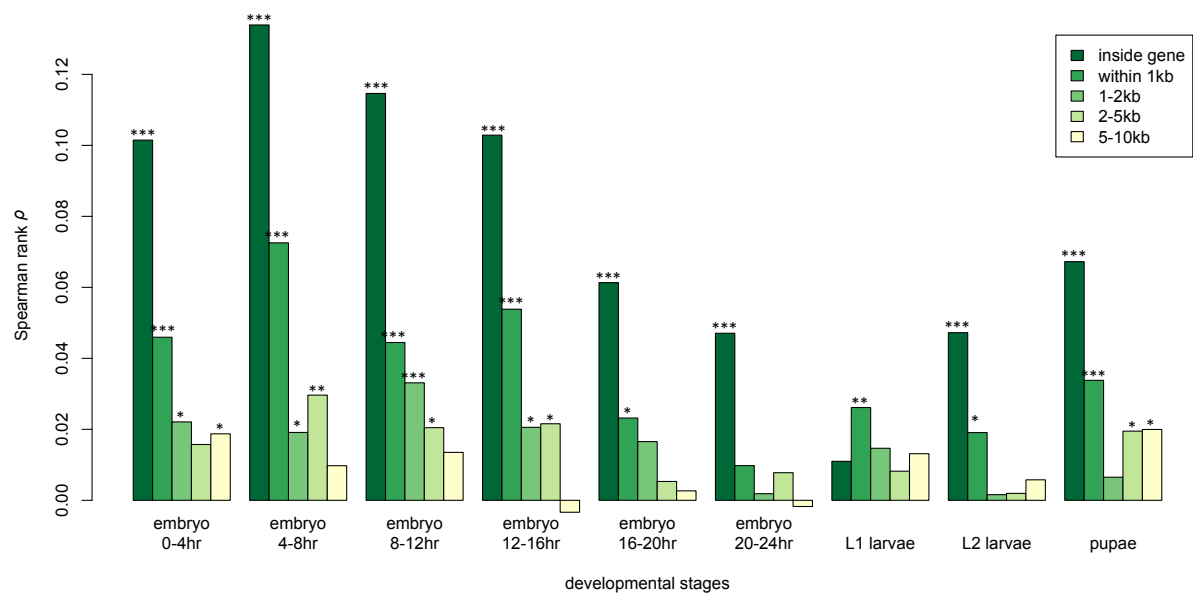

Supplement: S11 Fig — Partial correlation analyses were performed to account for the correlations between H3K9me3 density of genes and their local recombination rate. The analyses still found significant correlations between H3K9me3 density of a gene and the number of adjacent TEs within a specific window. In addition, the correlations decrease as the distance between the gene and the designed window increases. Notations for p-values are * (< 0.05), ** (< 0.01), *** (< 0.001). (PDF) [file pgen.1005269.s011.pdf]

A

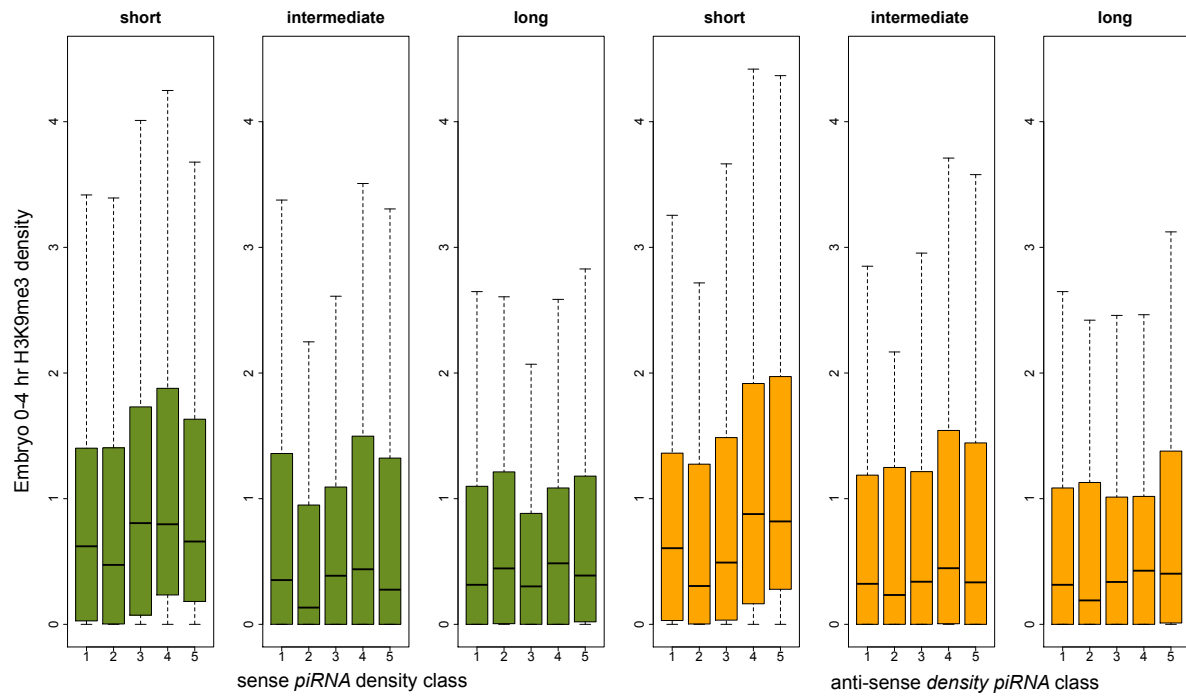

B

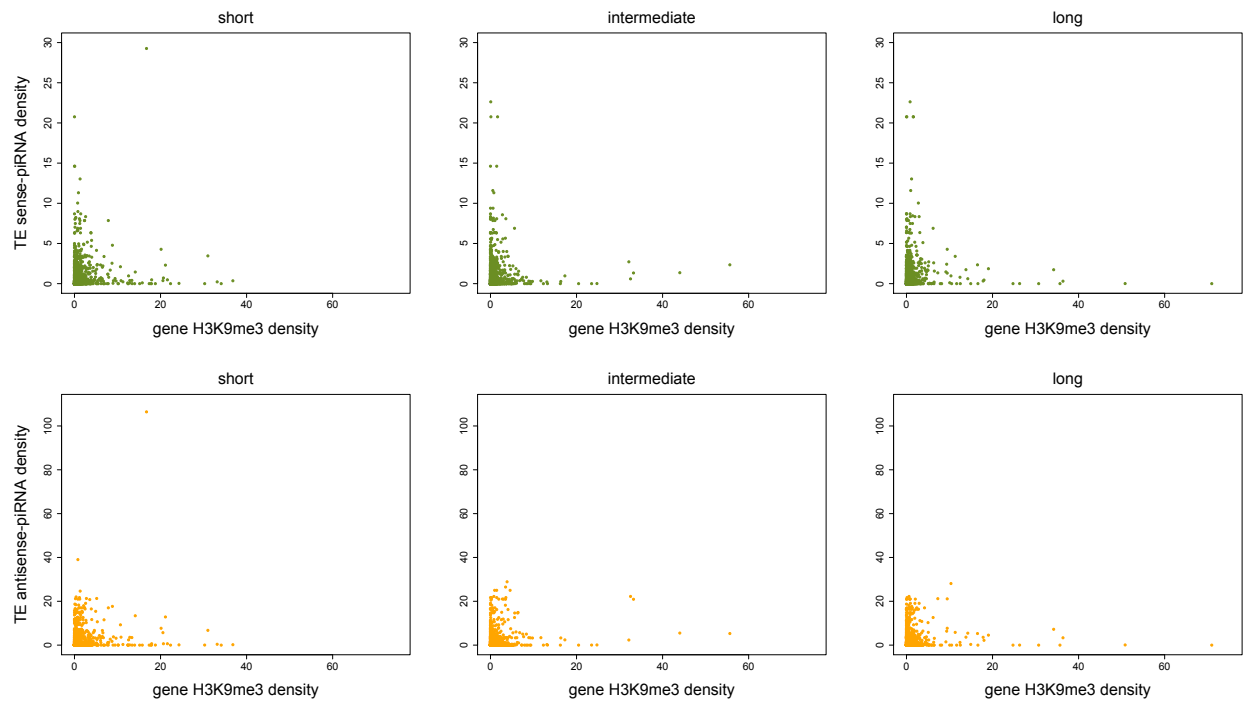

Supplement: S12 Fig — (A) Gene-TE pairs were classified into three equal–size groups according to their distance from the nearest TEs (short, intermediate, and long). TEs of these gene-TE pairs were categorized into five equal bins according to their piRNA density (1–5, from lowest to highest piRNA density). Figures are boxplots for genes whose nearest TEs are of different piRNA bins. Genes whose nearest TEs are targeted by higher piRNA density have higher H3K9me3 density and this trend is more pronounced for genes that are closer to TEs. The same data presented as scatter plots are shown in (B). (PDF) [file pgen.1005269.s012.pdf]

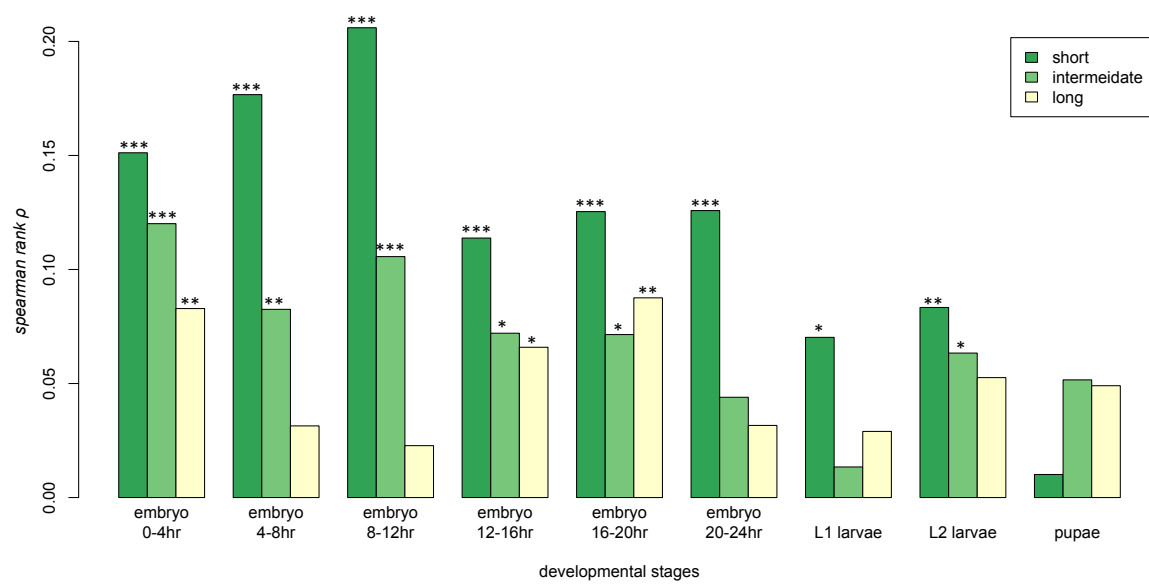

Supplement: S13 Fig — The correlations decrease as the distances between the gene and the TE increase (represented with different color bars). Notations for p-values are * (< 0.05), ** (< 0.01), *** (< 0.001). (PDF) [file pgen.1005269.s013.pdf]
