# Supplementary material for: The Role of piRNA-Mediated Epigenetic Silencing in the Population Dynamics of Transposable Elements in Drosophila melanogaster
Source: PLoS Genet. 2015 Jun 4;11(6):e1005269. doi: 10.1371/journal.pgen.1005269 (PMC4456100; doi:10.1371/journal.pgen.1005269)
Supplement: S1 Table — The H3K9me3 density of 1kb windows that are of different distance from TEs were compared to those of window that are 9-10kb away from TEs using Mann-Whitney U test. (PDF) [file pgen.1005269.s014.pdf]

	0-1kb		1-2kb		2-3kb		3-4kb		4-5kb	
**developmental stage**	***median***	***p-value***	***median***	***p-value***	***median***	***p-value***	***median***	***p-value***	***median***	***p-value***
**embryo 0-4hr**	2.6316	9.519E-05	2.2574	2.114E-03	1.9675	9.114E-03	1.6337	1.064E-01	1.8851	8.000E-02
**embryo 4-8hr**	6.7738	4.681E-09	4.7589	1.941E-03	4.4276	8.119E-03	3.8885	7.576E-02	3.8368	9.066E-02
**embryo 8-12hr**	3.5904	4.149E-05	3.4315	4.992E-04	2.8078	1.347E-02	2.3596	8.409E-02	2.0090	3.868E-01
**embryo 12-16hr**	5.8160	7.086E-07	5.0795	1.575E-04	4.3718	8.794E-04	3.9895	3.058E-02	4.1035	8.777E-03
**embryo 16-20hr**	4.1875	4.518E-05	3.6643	2.444E-03	3.2152	2.557E-02	2.4502	1.240E-01	2.5313	8.753E-02
**embryo20-24hr**	1.1186	6.242E-03	1.2096	3.931E-03	1.1196	1.278E-02	1.0854	3.314E-02	1.0225	5.784E-02
**L1 larva**	1.4219	3.001E-05	1.1507	1.139E-02	1.0497	1.265E-01	1.0328	2.268E-01	0.9980	3.059E-01
**L2 larva**	5.5370	2.210E-04	5.4851	6.972E-04	4.7067	5.627E-02	4.4303	1.908E-01	4.7577	1.467E-01
**Pupae**	1.4963	1.194E-03	1.3588	3.043E-03	1.2179	6.407E-02	1.0758	1.930E-01	1.1999	2.114E-01

	5-6kb		6-7kb		7-8kb		8-9kb		9-10kb
**developmental stage**	***median***	***p-value***	***median***	***p-value***	***median***	***p-value***	***median***	***p-value***	***median***
**embryo 0-4hr**	1.9222	7.097E-02	1.6023	5.613E-01	1.6340	6.230E-01	1.5938	6.398E-01	1.5441
**embryo 4-8hr**	3.3164	2.891E-01	3.0432	5.840E-01	3.1193	6.430E-01	2.7966	8.309E-01	3.0157
**embryo 8-12hr**	2.1448	4.204E-01	1.9883	6.403E-01	1.6762	8.295E-01	1.9688	9.364E-01	1.6719
**embryo 12-16hr**	4.4053	9.981E-03	3.6620	2.214E-01	3.5245	4.362E-01	3.6015	4.430E-01	3.1981
**embryo 16-20hr**	2.6818	1.714E-01	2.6245	2.381E-01	2.2213	3.870E-01	2.4635	4.420E-01	1.9718
**embryo20-24hr**	1.0689	4.457E-02	0.9664	3.214E-01	0.9952	3.653E-01	0.8284	6.441E-01	0.8585
**L1 larva**	0.9274	5.778E-01	0.8811	8.177E-01	0.8469	6.975E-01	0.9004	8.101E-01	0.8650
**L2 larva**	4.5326	3.052E-01	4.4402	1.999E-01	4.1809	3.752E-01	4.3704	4.293E-01	4.1762
**Pupae**	1.1956	4.450E-01	1.0109	7.630E-01	0.9800	8.947E-01	1.0336	9.301E-01	0.9761
