# Supplementary material for: The Role of piRNA-Mediated Epigenetic Silencing in the Population Dynamics of Transposable Elements in Drosophila melanogaster
Source: PLoS Genet. 2015 Jun 4;11(6):e1005269. doi: 10.1371/journal.pgen.1005269 (PMC4456100; doi:10.1371/journal.pgen.1005269)
Supplement: S2 Table — (PDF) [file pgen.1005269.s015.pdf]

developmental stage	H3K9me3 density vs distance to TE<br>*ρ*	*p-value*
**Embryo 0-4hr**	-0.1330	$< 10^{-16}$
**Embryo 4-8hr**	-0.1894	$< 10^{-16}$
**Embryo 8-12hr**	-0.1491	$< 10^{-16}$
**Embryo 12-16hr**	-0.1559	$< 10^{-16}$
**Embryo 16-20hr**	-0.1023	7.12E-09
**Embryo20-24hr**	-0.0661	1.88E-04
**L1 larvae**	-0.0399	2.43E-02
**L2 Larvae**	-0.0560	1.55E-03
**Pupae**	-0.0875	7.35E-07
