# Supplementary material for: The Role of piRNA-Mediated Epigenetic Silencing in the Population Dynamics of Transposable Elements in Drosophila melanogaster
Source: PLoS Genet. 2015 Jun 4;11(6):e1005269. doi: 10.1371/journal.pgen.1005269 (PMC4456100; doi:10.1371/journal.pgen.1005269)
Supplement: S3 Table — A small fraction of piRNAs was found mapped to genes. The piRNA densities of genes are significantly lower than the piRNA densities of TEs. Correlations between genic H3K9me3 density and piRNA density of genes/nearest TEs were estimated using Spearman Rank correlation test. Unlike the correlations between a gene’s H3K9me3 density and the piRNA density of its nearest gene, we did not observe positive correlations between a gene’s H3K9me3 density and its piRNA density. (PDF) [file pgen.1005269.s016.pdf]

	comparison of *piRNA* density			correlation with genic H3K9me3 density			
	median		*Mann-Whitney U test*	gene		TE	
	**gene**	**TE**	***p-value***	***ρ***	***p-value***	***ρ***	***p-value***
**sense *piRNA***	0.0537	0.256	$< 10^{-16}$	-0.180	$< 10^{-16}$	0.0428	1.6E-02
**anti-sense *piRNA***	0	0.186	$< 10^{-16}$	-0.0376	7.1E-05	0.0838	2.2E-06
