# Supplementary material for: The Role of piRNA-Mediated Epigenetic Silencing in the Population Dynamics of Transposable Elements in Drosophila melanogaster
Source: PLoS Genet. 2015 Jun 4;11(6):e1005269. doi: 10.1371/journal.pgen.1005269 (PMC4456100; doi:10.1371/journal.pgen.1005269)
Supplement: S4 Table — The correlations were performed for all euchromatic genes included in the analyses (all genes) as well as for genes that have TEs within 10kb (genes with TE in 10kb). (PDF) [file pgen.1005269.s017.pdf]

| H3K9me3 | expression | all genes | | genes with TE in 10kb | |
|---|---|---|---|---|---|
| | | ***ρ*** | ***p-value*** | ***ρ*** | ***p-value*** |
| **embryo 0-4hr** | **embryo 0-2hr** | -1.308E-01 | < 10-16 | -1.383E-01 | 2.4E-09 |
| | **embryo 2-4hr** | -1.521E-01 | < 10-16 | -1.719E-01 | 5.0E-15 |
| **embryo 4-8hr** | **embryo 4-6hr** | -1.371E-01 | < 10-16 | -1.716E-01 | 2.7E-15 |
| | **embryo 6-8hr** | -1.276E-01 | < 10-16 | -1.458E-01 | 6.1E-12 |
| **embryo 8-12hr** | **embryo 8-10hr** | 1.157E-02 | 3.0E-01 | -1.368E-02 | 5.1E-01 |
| | **embryo 10-12hr** | 2.962E-03 | 7.9E-01 | -3.489E-02 | 8.9E-02 |
| **embryo 12-16hr** | **embryo 12-14hr** | -2.261E-01 | < 10-16 | -2.329E-01 | < 10-16 |
| | **embryo 14-16hr** | -2.479E-01 | < 10-16 | -2.463E-01 | < 10-16 |
| **embryo 16-20hr** | **embryo 16-18hr** | -3.898E-02 | 2.6E-04 | -3.895E-02 | 4.7E-02 |
| | **embryo 18-20hr** | 1.576E-02 | 1.4E-01 | 1.965E-02 | 3.1E-01 |
| **embryo20-24hr** | **embryo 20-22hr** | -2.935E-02 | 5.7E-03 | -3.959E-04 | 9.8E-01 |
| | **embryo 22-24hr** | -4.742E-02 | 6.9E-06 | -1.141E-02 | 5.5E-01 |
| **L1 larva** | **L1 larva** | -1.397E-01 | < 10-16 | -9.866E-02 | 3.9E-07 |
| **L2 larva** | **L2 larva** | -9.249E-02 | < 10-16 | -7.793E-02 | 6.5E-05 |
| **Pupae** | **pupae 2d postWPP** | -8.455E-02 | < 10-16 | -5.419E-02 | 1.9E-03 |
