# Supplementary material for: The Role of piRNA-Mediated Epigenetic Silencing in the Population Dynamics of Transposable Elements in Drosophila melanogaster
Source: PLoS Genet. 2015 Jun 4;11(6):e1005269. doi: 10.1371/journal.pgen.1005269 (PMC4456100; doi:10.1371/journal.pgen.1005269)
Supplement: S5 Table — Genes are categorized according to their distance from the nearest TE (up to 10kb). The expression levels of each category genes that have TEs nearby are compared to expression levels of genes without TEs nearby using Mann-Whitney U test. (PDF) [file pgen.1005269.s018.pdf]

	median gene expression level of genes						*Mann-Whitney U test p-value*				
	with TE nearby					without TE					
**developmental stage**	**in gene**	**1kb**	**2kb**	**5kb**	**10kb**	**nearby**	**in gene**	**1kb**	**2kb**	**5kb**	**10kb**
**embryo 0-2hr**	21.5	25	27	28	29	33	1.8E-08	9.4E-09	6.2E-06	9.9E-06	3.1E-03
**embryo 2-4hr**	12	14	14	15	16	18	1.5E-05	1.4E-05	1.6E-05	6.8E-05	5.2E-03
**embryo 4-6hr**	12	14	14	15	16	18	5.1E-06	2.0E-07	5.0E-07	1.3E-05	5.8E-03
**embryo 6-8hr**	14	14	15	15	16	18	4.5E-04	5.7E-06	4.4E-06	3.7E-05	1.2E-02
**embryo 8-10hr**	14	14	15	15	16	17	1.5E-03	9.7E-05	1.0E-04	1.3E-04	8.1E-03
**embryo 10-12hr**	12	13	13.5	14	14	16	3.6E-05	1.9E-05	3.5E-05	1.6E-04	5.9E-03
**embryo 12-14hr**	14	13	13	13	13	15	6.6E-02	7.5E-03	6.6E-03	2.0E-03	1.1E-02
**embryo 14-16hr**	13	12	12	12	12	14	1.5E-01	1.1E-02	4.9E-03	8.9E-03	3.1E-02
**embryo 16-18hr**	13	13	13	12	13	13	5.2E-01	6.9E-01	5.6E-01	3.9E-01	5.2E-01
**embryo 18-20hr**	13	12	12	12	12	11	3.2E-01	1.6E-01	2.8E-01	3.3E-01	1.1E-01
**embryo 20-22hr**	11	12	12	12	12	12	3.2E-01	7.7E-01	5.6E-01	3.4E-01	1.1E-01
**embryo 22-24hr**	13	13	13	13	13	13	5.2E-01	4.6E-01	3.1E-01	1.1E-01	5.8E-02
**L1 larva**	8	9	10	11	11	11	8.2E-06	3.1E-02	1.6E-01	6.0E-01	4.8E-01
**L2 larva**	6	8	8	10	10	11	3.3E-18	6.6E-08	6.2E-06	4.2E-03	3.8E-01
**L3 larva 12hr old**	6	7	8	9	9	9.5	1.1E-17	1.9E-05	1.7E-03	2.6E-01	3.8E-01
**L3 larva puff stage 1-2**	5	7	7	7	8	8	1.2E-09	1.2E-02	5.5E-02	8.8E-01	2.6E-01
**L3 larva puff stage 3-6**	8.5	9	9	10	10	10	3.6E-07	6.8E-03	3.7E-02	4.4E-01	6.8E-01
**L3 larva puff stage 7-9**	10	11	11	12	12	12	7.8E-06	3.7E-03	2.2E-02	2.3E-01	9.6E-01
**white prepupae new**	11	11	11	11	11	11	1.9E-02	7.2E-02	1.8E-01	4.7E-01	6.2E-01
**white prepupae 12hr**	12	12	13	13	13	13	2.4E-03	3.3E-02	1.3E-01	2.2E-01	9.0E-01
**white prepupae 24hr**	13	13	13	13	14	15	8.5E-04	2.0E-04	1.8E-03	5.3E-03	1.3E-01
**pupae 2d postWPP**	14	13	14	14	14	14	5.5E-02	1.0E-02	2.9E-02	2.3E-02	2.1E-01
**pupae 3d postWPP**	10	10	10	10	11	11	4.0E-03	1.7E-03	4.3E-03	7.1E-03	2.6E-01
**pupae 4d postWPP**	8	8	9	9	10	10	2.1E-04	7.0E-05	2.7E-03	7.7E-03	3.7E-01
**adult fembryo ale 1 day**	9	11	12	12	13	14	4.3E-14	2.0E-08	8.8E-06	8.0E-05	3.3E-02
**adult fembryo ale 5 days**	11	15	16	17	18	20	4.1E-13	1.6E-07	5.3E-05	7.6E-04	5.4E-02
**adult fembryo ale 30 days**	11	15	16	18	19	21	1.2E-14	7.3E-09	1.0E-05	2.0E-04	2.1E-02
**adult male**	8	10	11	11	12	13	6.5E-12	1.1E-05	1.6E-03	1.6E-02	4.1E-01
**adult male 5 days**	8	11	12	12	13	14	6.7E-14	1.3E-05	6.2E-03	4.8E-02	6.5E-01
**adult male 30 days**	8	10	11	12	12	13	2.5E-14	4.1E-07	8.7E-04	8.6E-03	2.7E-01
