# Supplementary material for: The Role of piRNA-Mediated Epigenetic Silencing in the Population Dynamics of Transposable Elements in Drosophila melanogaster
Source: PLoS Genet. 2015 Jun 4;11(6):e1005269. doi: 10.1371/journal.pgen.1005269 (PMC4456100; doi:10.1371/journal.pgen.1005269)
Supplement: S8 Table — (PDF) [file pgen.1005269.s021.pdf]

window size with TE	no. significant gene			***FET test***	
	**both**	**female**	**male**	***p-value***	**odds ratio**
**in gene**	18	41	40	8.7E-09	10.017
**1kb**	22	48	51	2.5E-11	11.324
**2kb**	23	50	53	1.9E-13	13.998
**5kb**	27	78	79	1.6E-12	8.465
**10kb**	46	136	131	< 1E-16	9.770
