# Supplementary material for: The Role of piRNA-Mediated Epigenetic Silencing in the Population Dynamics of Transposable Elements in Drosophila melanogaster
Source: PLoS Genet. 2015 Jun 4;11(6):e1005269. doi: 10.1371/journal.pgen.1005269 (PMC4456100; doi:10.1371/journal.pgen.1005269)
Supplement: S9 Table — (PDF) [file pgen.1005269.s022.pdf]

developmental stage	H3K9me3 density vs TE frequency *ρ*	*p-value*
**Embryo 0-4hr**	-0.0831	2.0E-02
**Embryo 4-8hr**	-0.1516	2.0E-05
**Embryo 8-12hr**	-0.0959	7.1E-03
**Embryo 12-16hr**	-0.1209	6.8E-04
**Embryo 16-20hr**	-0.1351	1.5E-04
**Embryo20-24hr**	-0.1331	1.8E-04
**L1 larvae**	-0.0617	8.3E-02
**L2 Larvae**	-0.1110	1.8E-03
**Pupae**	-0.0369	3.0E-01
