# Supplementary material for: The Role of piRNA-Mediated Epigenetic Silencing in the Population Dynamics of Transposable Elements in Drosophila melanogaster
Source: PLoS Genet. 2015 Jun 4;11(6):e1005269. doi: 10.1371/journal.pgen.1005269 (PMC4456100; doi:10.1371/journal.pgen.1005269)
Supplement: S10 Table — TEs of gene-TE pairs were classified into those that are observed (Observed) and not observed (Not observed) in a North American D. melanogaster population. We performed two-way ANOVA to test whether, when accounting for the effect of TE families, the H3K9me3 density of genes varies according to the population frequencies of their nearest TEs (model: H3K9me3 density ~ observed/not + TE family + observed/not * TE family). Our analysis showed that TE frequency has a significant effect on variation of genic H3K9me3 density at six developmental stages. We used two ways to infer the directionality of the influence of TE frequency on genic H3K9me3 density while taking into account the influence of TE families. According to the results of ANOVA, the interaction terms are not significant for all except one developmental stage at which TE frequency has a significant effect on H3K9me3 density (embryo 4–8 hr, 8–12 hr, 16–20 hr, 20–24 hr, and L2 larvae), suggesting that the directionality of the differences in H3K9me3 density (i.e. “Not observed” genes have higher H3K9me3 density) is consistent across TE families. Second, we performed linear regression to examine the signs for the coefficients of TE frequency (categorical “observed/not” was changed to numerical “TE frequency”). For developmental stages at which the influence of TE frequency on genic H3K9me3 density is significant in the ANOVA analysis, the regression coefficients of TE frequency have negative signs except for one developmental stage. It is worth noting that, because of the large number of TE families (50 families were included in this analysis), few terms are significant in the linear regression analysis due to the reduction in degrees of freedom. Alternatively, we treated the effect of TE family on genic H3K9me3 density as random and performed mixed linear model analysis. The coefficients of TE frequency are significant for four developmental stages and they all have negative signs. Our analyses suggest that, [file pgen.1005269.s023.pdf]

	ANOVA TE family as fixed effect			Two sample *Mann-Whitney U test* for genes with nonzero H3K9me3 density			linear regression TE family as fixed effect		mixed linear regression TE family as random effect	
	p-value			genic H3K9me3 median			coefficient of TE frequency		coefficient of TE frequency	
	**TE frequency**	**TE family**	**interaction**	**Not observed**	**Observed**	***p-value***	**coefficient**	**sign**	**coefficient**	***p-value***
**Embryo 0-4hr**	3.7E-01	1.4E-01	3.4E-02	0.964	0.918	6.1E-02	-5.8E-01	-	-0.841	1.3E-01
**Embryo 4-8hr**	2.5E-06	4.8E-07	9.1E-01	1.092	0.570	5.0E-06	-5.9E-01	-	-0.899	2.6E-03
**Embryo 8-12hr**	2.4E-03	5.1E-06	7.5E-01	1.514	1.233	5.3E-03	-3.5E-01	-	-0.757	3.1E-02
**Embryo 12-16hr**	1.1E-02	1.3E-04	1.5E-02	1.047	0.707	8.4E-03	3.7E-01	+	-0.426	1.6E-01
**Embryo 16-20hr**	3.4E-02	8.8E-05	2.2E-01	5.375	4.118	5.7E-03	-1.1E-03	-	-0.275	1.5E-01
**Embryo 20-24hr**	5.0E-03	5.5E-02	6.1E-02	2.984	2.661	1.7E-03	-4.2E-01	-	-0.385	1.3E-02
**L1 larvae**	5.4E-01	3.5E-03	4.4E-02	1.354	1.251	2.9E-01	7.6E-02	+	-0.100	5.7E-01
**L2 Larvae**	6.3E-04	8.6E-03	3.4E-01	8.655	7.846	5.4E-03	-5.8E-01	-	-0.438	1.1E-02
**Pupae**	7.8E-01	7.0E-01	2.8E-02	1.106	1.120	5.5E-01	-1.4E-01	-	0.139	4.4E-01
