# Supplementary material for: The Role of piRNA-Mediated Epigenetic Silencing in the Population Dynamics of Transposable Elements in Drosophila melanogaster
Source: PLoS Genet. 2015 Jun 4;11(6):e1005269. doi: 10.1371/journal.pgen.1005269 (PMC4456100; doi:10.1371/journal.pgen.1005269)
Supplement: S11 Table — Terms that are not selected in the backward AIC selection analysis are not included in the final regression model (denoted as “NA”). See Materials and Methods for regression models used. (PDF) [file pgen.1005269.s024.pdf]

developmetal stage	H3K9me3 density (per bp) beta	H3K9me3 density (per bp) *p-value*	H3K9me3 density^2 beta	H3K9me3 density^2 *p-value*	recombination rate (cM/Mbp) beta	recombination rate (cM/Mbp) *p-value*	recombination rate^2 beta	recombination rate^2 *p-value*
**Embryo 0-4hr**	-0.0300	6.1E-03	8.20E-04	3.9E-02	-0.1163	1.0E-06	0.0138	8.9E-06
**Embryo 4-8hr**	-0.0165	9.7E-06	1.62E-04	4.6E-04	-0.1147	1.1E-06	0.0139	7.3E-06
**Embryo 8-12hr**	-0.0129	5.7E-05	1.12E-04	1.3E-03	-0.1188	5.0E-07	0.0143	4.1E-06
**Embryo 12-16hr**	-0.0148	1.3E-03	1.51E-04	1.7E-02	-0.1161	9.7E-07	0.0139	7.7E-06
**Embryo 16-20hr**	-0.0097	6.2E-05	6.85E-05	2.1E-03	-0.1185	5.5E-07	0.0142	4.5E-06
**Embryo 20-24hr**	-0.0257	1.1E-04	5.63E-04	2.1E-03	-0.1162	8.9E-07	0.0139	7.1E-06
**L1 larvae**	-0.0189	2.2E-02	NA	NA	-0.1160	1.1E-06	0.0138	9.9E-06
**L2 Larvae**	-0.0095	6.5E-04	1.01E-04	2.1E-02	-0.1206	3.9E-07	0.0145	3.1E-06
**Pupae**	-0.0503	1.1E-02	6.10E-03	1.0E-02	-0.1179	7.6E-07	0.0141	6.7E-06

	no. of dev. stages beta	no. of dev. stages *p-value*	no. of dev. Stages^2 beta	no. of dev. Stages^2 *p-value*	recombination rate (cM/Mbp) beta	recombination rate (cM/Mbp) *p-value*	recombination rate^2 beta	recombination rate^2 *p-value*
**all dev. Stages**	-0.0787	5.7E-04	0.0062	4.3E-02	-0.1160	7.9E-07	0.014033	5.22E-06
