# Supplementary material for: The Role of piRNA-Mediated Epigenetic Silencing in the Population Dynamics of Transposable Elements in Drosophila melanogaster
Source: PLoS Genet. 2015 Jun 4;11(6):e1005269. doi: 10.1371/journal.pgen.1005269 (PMC4456100; doi:10.1371/journal.pgen.1005269)
Supplement: S14 Table — This table summarizes, for 100 randomly selected sets of genes that have no TEs within 10kb, the proportion of genes with significant (one-tailed p-value < 0.05) differential expression between two randomly partitioned groups of alleles (see Materials and Methods). This was used to assess the false positive rate for the permutation procedure. (PDF) [file pgen.1005269.s027.pdf]

**window size with TE**	female				male			
	minimum	median	maxmium	mean	minimum	median	maxmium	mean
**in gene**	0.0277	0.0453	0.0730	0.0475	0.0252	0.0479	0.0831	0.0471
**1kb**	0.0267	0.0457	0.0686	0.0459	0.0248	0.0457	0.0686	0.0456
**2kb**	0.0281	0.0468	0.0640	0.0471	0.0250	0.0460	0.0671	0.0457
**5kb**	0.0345	0.0479	0.0633	0.0478	0.0297	0.0460	0.0623	0.0464
**10kb**	0.0369	0.0472	0.0585	0.0475	0.0364	0.0477	0.0575	0.0473
